# The Poisson distribution model fits UMI-based single-cell RNA-sequencing data

**DOI:** 10.21203/rs.3.rs-2517698/v1

**Published:** 2023-02-06

**Authors:** Yue Pan, Justin T. Landis, Razia Moorad, Di Wu, J.S. Marron, Dirk P. Dittmer

## Abstract

**Background:** Modeling of single cell RNA-sequencing (scRNA-seq) data remains challenging due to a high percentage of zeros and data heterogeneity, so improved modeling has strong potential to benefit many downstream data analyses. The existing zero-inflated or over-dispersed models are based on aggregations at either the gene or the cell level. However, they typically lose accuracy due to a too crude aggregation at those two levels. **Results:** We avoid the crude approximations entailed by such aggregation through proposing an Independent Poisson Distribution (IPD) particularly at each individual entry in the scRNA-seq data matrix. This approach naturally and intuitively models the large number of zeros as matrix entries with a very small Poisson parameter. The critical challenge of cell clustering is approached via a novel data representation as Departures from a simple homogeneous IPD (DIPD) to capture the per-gene-per-cell intrinsic heterogeneity generated by cell clusters. Our experiments using real data and crafted experiments show that using DIPD as a data representation for scRNA-seq data can uncover novel cell subtypes that are missed or can only be found by careful parameter tuning using conventional methods. **Conclusions:** This new method has multiple advantages, including (1) no needfor prior feature selection or manual optimization of hyperparameters; (2) flexibility to combine with and improve upon other methods, such as Seurat. Another novel contribution is the use of crafted experiments as part of the validation of our newly developed DIPD-based clustering pipeline. This new clustering pipeline is implemented in the R (CRAN) package *scpoisson* .

### Background

Single cell RNA-sequencing (scRNA-seq) estimates the transcriptome at the individual cell level. ScRNA-seq can directly measure cell-to-cell heterogeneity, which is more challenging using bulk RNA sequencing. First applied in 2009 [1], scRNA-seq has become the preferred tool to identify cell sub-populations and to investigate cellular heterogeneity [2, 3, 4, 5, 6, 7], gene regulatory networks [8, 9], stochastic fluctuations in transcription [10, 11], and so on. Due to the unique features of the data distribution in scRNA-seq, it's essential to develop statistical methods which accurately model scRNA-seq data for many important downstream analyses including differential expression analysis and clustering of cells.

Existing methods typically model the scRNA-seq data at the gene level for differential expression analysis to find biomarkers, and at the sample level for clustering of cells to find cell subtypes; however they typically lose accuracy due to a too crude aggregation at those two levels. This aggregation has led to attempts to explicitly model the apparent resulting zero-inflation or over-dispersion. We propose more precisely addressing these issues by modeling the distribution of each individual entry of the data matrix. A major challenge is that scRNA-seq data typically contain a large number of zero counts for gene/cell combinations (often exceeding 90%) [12]. This is due to both biological reasons that some genes are only expressed in a cluster of cells, and technical limitations such as low RNA capture rates, low efficiency library construction, cell disintegration and RNA degradation. There also exists a severe threshold effect in detection sensitivity of gene expression in scRNA-seq. Typically higher expressed genes in a cell tend to have a higher probability to be detected [13, 14, 4, 15]. These characteristics can lead to large discrepancies among sequencing libraries for different cells, i.e. batch effects, and render many global normalization approaches ineffective. Various approaches have been proposed to address barriers that limit the interpretation of scRNA-seq data [16, 17, 18, 19, 20, 21, 22, 23]. On the "wet-bench" side, unique molecular identifier (UMI) was introduced [24]. UMI reduces biases introduced by the extreme signal amplification that is necessary for scRNA-seq. Some researchers have argued that if the UMI technology works properly, there is no need to account for zero-inflation [25, 22, 26]. This is an encouraging perspective; however, these classical probability models are again only crude aggregations focusing on either cells or genes.

To improve the accuracy of statistical modeling and gain more precise inference, we propose the novel and principled approach of studying individual entries of the gene-by-cell matrix. This approach is based on the Independent Poisson Distribution (IPD) statistical framework, where every gene in each cell follows its own Poisson distribution. Working with such a model is challenging because the maximum likelihood estimate of each Poisson parameter is simply the corresponding count, which is too noisy to be useful. To solve this problem which presents for the validation of the IPD model we first start with several biologically homogeneous data sets derived from single clonal cell lines [27]. Next, we perform parameter estimation using generalized principal component analysis (GLM-PCA) [25] as a noise reduction method. While this approach has clear potential to eliminate noise when keeping important biological signals, it is challenging in most applications because the critical number of GLM-PCA components is not known. However, a fundamental exception to this principal nicely arises in the validation of the IPD model. This is because if we can find (by trial and error) a number of components which result in a fit of the standard univariate Poisson distribution to collections of matrix entries having very similar parameters, then the goodness of fit of the IPD is verified. The fit of *Poissoneity* to sets of similar matrix entries is studied using Quantile-to-Quantile plots (Q-Q plots), together with simulated envelopes indicating natural variation, in addition to over-dispersion and zero-inflation hypothesis tests.

Based on this newly proposed IPD framework which focuses on individual entries of the scRNA-seq data matrix, we further develop procedures based on the computation of Departure from the IPD (DIPD) as a data representation to replace the scRNA-seq count data by the logistic transformation of probabilities of Departure to ensure modeling accuracy and to effectively deal with zeros. The output will be a data matrix of the same dimension of scRNA-seq with continuous values. This enables our development of other new computational approaches including clustering and other downstream tasks through the novel concept of DIPD. The DIPD is initialized by a rough two-way parameter approximation of the data. Next, different cell types are captured by departures from the naïve two-way approximation. Then the data is bisected using Poisson departure as the distance measure. The clustering algorithm terminates, when there is no significant deviation from Poissoneity for any cell group. For some data [28] this approach gives results similar to those using other pipelines. For others [29] it shows an improvement. Overall, for additional downstream tasks, the DIPD matrix is proposed as a new data representation (*model departure*).

In sum, the IPD statistical framework has the potential to capture meaningful biological properties at a higher resolution than prior normalization methods, without the need for more complicated probability distributions. We demonstrate the usefulness of model departure DIPD as a novel data representation by conducting downstream analysis, such as clustering of cells. Our newly developed DIPD-based clustering pipeline is validated in multiple experimental data. Another important contribution of this paper is the use of the novel method called *crafted experiments* for the comparison of the DIPD with other methods in a principled way. While we demonstrate the value of our proposed model departure data representation for clustering, we anticipate it will be useful for additional downstream tasks, such as differential expression analysis, gene set tests and trajectory analysis, because it provides a useful replacement of the conventional data matrix.

### Results

#### Validation of Poissoneity for scRNA-seq data

Poissoneity postulates that each matrix entry (gene by cell) comes from an independent Poisson distribution. As stated in Methods, the Poisson parameter for each matrix entry can be estimated using GLM-PCA [25]. However, the success of that estimation requires a good choice of the number of latent vectors $L$, which is generally quite challenging. The model validation context we consider here allows an unusual approach to that challenge. In particular, finding a value of $L$ which gives a good fit of the resulting IPD model establishes its validity. That goodness of fit is quantified here using both Q-Q envelope visualization and formal hypothesis testing.

To study the Poissoneity of scRNA-seq data, we first explore the simplest case: cells picked at random from a clonal cell line processed as a single batch (Plate 3 [27]) with $L = 10$ (for the reasons given in section Methods). In Fig. 1, panels a, b and c display the distribution histograms. For a given Poisson parameter $\lambda = 0.5$, $\lambda = 2$ or $\lambda = 20$, the gold bars represent distributions based on 200 aggregated UMI

entries with the estimated Poisson parameters closest to $\lambda$. Their distributions approximately follow the theoretical Poisson($\lambda$) distributions (gray bars). In contrast, the distributions from entries of genes whose gene averages are closest to $\lambda$ (blue bars), do not.

Fig. 1 panels d, e and f show the corresponding Q-Q envelope plots (see Methods). These provide an alternative display of the distribution of the data. For all three $\lambda$ choices, the gold lines (based on aggregated matrix entries) are within the gray envelopes of variation, indicating good fits using the Poisson distributions. The gene-level entries (blue line) do not lie within the Q-Q envelope indicating a poor Poisson fit. Furthermore, the manner in which the blue curves leave the envelope show both the typically expected zero-inflation (departure below on the left) and over-dispersion (departing above on the right). This demonstrates that individual raw UMI count entries follow Poisson distributions, but genes, whose averages are often used for normalization, do not.

The hypothesis testing (based on aggregated matrix entries) have p-values $p = 0.155$ for $\lambda = 0.5$, $p = 0.056$ for $\lambda = 2$ and $p = 0.004$ for $\lambda = 20$ from over-dispersion tests; and $p = 0.278$ for $\lambda = 0.5$, $p = 0.389$ for $\lambda = 2$ and $p = 1.000$ for $\lambda = 20$ from the zero-inflation tests. These are consistent with the visual representation. An exception is $\lambda = 20$ (for the over-dispersion test). Here, (Fig. 1, panel f) the UMI-based individual entries distribution (gold) goes outside the gray variation envelope at the top for high values. This is due to a sampling effect. Relatively few matrix entries have parameter estimates close to $\lambda = 20$, i.e. sampled entries come from a mixture of Poissons due to variation in the underlying parameters. If we decreased the number of aggregated entries to 100, then the over-dispersion test is not significant ($p = 0.129$) even when $\lambda = 20$. This result indicates a high quality of fit for the IPD statistical framework and is consistent with the notion that UMI count-based scRNA-seq data can be modeled by independent Poisson distributions at the individual gene-cell entry level.

### Further goodness of fit investigations

Next, we use these goodness of fit tools (for matrix entries with similar Poisson parameters) to study batch variation (Fig. 2). Each plate represents a technical replicate (batch) or different biological condition as defined in Methods. Within each plate, we took $\lambda$ ranging from 0.1 to 20, on 200 aggregated matrix entries (Poisson parameters are again estimated using GLM-PCA [25] with $L = 10$) to test for Poissoneity using Q-Q envelope plots and hypothesis testing. Based on this extended data we find that: first, UMI data fall within the variation envelope (gray lines) on Q-Q envelope plots, suggesting that the Poisson distribution fit the matrix entries; second, inflated zeros are not detectable for UMI entries based on zero-inflation tests ($p > 0.05$); third, no over-dispersion is detectable for UMI entries based on dispersion hypothesis testing ($p > 0.05$). The exception is $\lambda = 20$, which can be explained as a mixture of Poisson as discussed above.

One of the experiments deliberately violated the single cell assumption in a novel direction. Plate 8 (green) had two cells per well, i.e. per library. It shows over-dispersion at $L = 10$ ($p = 0.049$ when $\lambda = 5$ and $p = 0.007$ when $\lambda = 20$). This is consistent with the experimental design. It had a stronger signal for low abundance transcripts as twice as much RNA was present per well, which resulted in more biological variation. This different signal to noise ratio is handled by increasing $L$ to 15. Compare the light ($L = 10$) and dark green ($L = 15$) curves in Fig. 2 panels e and f. At $L = 15$, the curves are within the envelopes and the over-dispersion tests have p-values $p = 0.882$ when $\lambda = 5$ and $p = 0.087$ when $\lambda = 20$, indicating no over-dispersion.

Another experiment has an equal mixture of two different cell lines (Plates 5A and 6A). In Fig. 2 panel f, the Q-Q envelope plot shows strong deviations at the bottom for low values at $L = 10$ when $\lambda = 20$ (orange curve; $p < 0.001$ for the over-dispersion test even decrease the number of selected entries to 100). This is because for this more heterogeneous data set, $L = 10$ components are inadequate to capture the biological variation. The fit is improved by increasing the number of latent vectors to $L = 20$ (the dark red curve; $p = 0.054$ for the over-dispersion test when $\lambda = 20$). These experiments show that deviations from cell homogeneity, either as a violation of the single cell assumption or as a result of a mixture of cells with different transcription profiles can be detected as departures from the IPD model. This property can be compensated for by increasing the number of latent variables $L$ or it can be exploited by a clustering algorithm using Poisson model departure as the distance metric. This algorithm is described below.

### Poisson departure data representation

Here, we introduce a novel data representation (DIPD) based on a departure from the IPD. The initial step is based on a crude two-way parameter approximation, where variation across cells is modeled by a cell-level parameter, and variation across genes is modeled by a gene-level parameter (as defined in equation (3)). This initialization step in itself does not appropriately account for cell heterogeneity. In the next step, the interesting cell structure is captured by departures from the naïve two-way approximation in both genes and cells, and the original count matrix is replaced by a Poisson departure matrix. In the departure matrix, each entry is quantified by the relative location of that original count with respect to the tentative Poisson distribution, whose parameter comes from the initial two-way approximation. The departure measure is captured by a Poisson Cumulative Distribution Function (CDF), which leaves the unexpectedly small counts nearly 0 and unusually large counts close to 1. Next, the departure measure is put on a more statistically amenable scale using the logit function. As a result, unexpectedly large counts give large positive values and unexpectedly small counts give large negative values.

Fig. 3 shows the heatmap visualizations (two cell lines data defined in the following section) based on DIPD (panel a) or Seurat after normalization and scaling (panel b) as data representations. Note the different scale ranges. The black lines in the sidebars depicted the top 2,000 most variable genes identified by Seurat. The DIPD-based representation kept all genes, as they may become relevant for defining sub-clusters, and also may be associated with important meta information. Such meta-information may include drug susceptibility or the availability of a clinical or histochemical assay to measure protein expression. The opportunity to identify genes of high clinical value is lost in approaches that select features based on statistical properties alone. In this simple case with two distinct cell lines, both representations perform similarly as depicting the differentially expressed (DE) genes between the two cell lines. We will show that the DIPD-based data matrix outperforms Seurat normalized counts as a novel data representation in a later section.

#### Cell type clustering based on Poisson departure

A major application of this data representation is cell clustering using DIPD. This can be used directly as input into other algorithms. It also opens the possibility for a novel clustering algorithm, as illustrated in Fig. 4. This algorithm, referred to as *Hclust-Departure*, operates as follows: Starting with the UMI count matrix ($UMI$), a very crude two-way parameter approximation (more details in Methods) is used to estimate Poisson parameters ($\tilde{\Lambda}$). Cell heterogeneity is not assumed at this step. Next, each UMI count is replaced by the DIPD ($D$) measure from the naïve model. This DIPD-based matrix serves as the input for the clustering step. Clustering with $k = 2$ is applied and the two-way approximation and DIPD-based data matrix is recalculated separately for each of the two subclusters. This process is repeated until (a) the split is no longer statistically significant; (b) the maximum allowable number of splitting steps is reached; or (c) any current cluster has less than 10 cells. Statistical significance is calculated using Sigclust2 [30]. For a homogeneous cluster of cells, all the departure entries ($D$) are similar, and therefore Sigclust2 should not find significant clusters.

To investigate the performance of *Hclust-Departure*, we compared it with a commonly used package, Seurat (version 3.1.1) [31].

##### *Single clonal cell line*

First, homogeneous data from a single clonal cell line (Plate 3) is tested [27]. There are no known clusters. This data serves as a negative control because the cells have been maintained under optimal growth conditions to minimize variations within the cell population. Applying *Hclust-Departure* to the DIPD-based matrix resulted in no significant splits ($p = 0.933$), consistent with the experimental design (panel a in Additional file 1). Seurat also identified only one cluster (resolution parameter 0.8, panel b in Additional file 1).

##### *Two cell lines, equal mixture*

Combining the data from two clonal cell lines (Plates 5A and 6A) in an equal mixture provided a positive control, as the two cancer cell lines were from independent patients, but of the same lineage [27]. *Hclust-Departure* resulted in two clusters, consistent with the known cell lines. Seurat also identified two clusters under the default setting (resolution parameter 0.8) as expected (Fig. 3).

*Three cell lines, unequal mixture*

Next, we applied *Hclust-Departure*, to data comprised of three mixture cell lines, at a ratio of 1:3:6 [32]. *Hclust-Departure* identified three clusters (panel a in Additional file 2). Using the default setting, Seurat identified 7 clusters. By tuning the Seurat resolution parameter from the default 0.8 to 0.1, overfitting was resolved (panel b in Additional file 2) and both approaches identified the three biologically defined clusters.

*Multiple cell lineages, unequal mixture*

To increase the complexity of the data further, data from the lymphoid organs of a mouse [28] was analyzed. These represent the complex lineages and populations of the hematopoietic system: T and B cells, which mediate the adaptive immune response, as well as dendritic cells (DCs), macrophages, mast cells, etc., which mediate the innate immune response as well as red blood cells (erythrocytes). Within each of these broad classes, multiple subclasses are recognized.

The results are visualized using t-distributed Stochastic Neighbor Embedding (t-SNE) [33] and Uniform Manifold Approximation and Projection (UMAP) [34] in Fig. 5 panels a, c and panels b, d. *Hclust-Departure* (panels a and b) is used without dimensionality reduction or feature selection. Seurat (panels c and d) is applied using the top 2,000 most variable features as defined by default. The cell type labels are manually assigned to each cluster using known lineage markers. The clusters discovered by *Hclust-Departure* are consistent with those identified by Seurat. Furthermore, *Hclust-Departure* identifies several significant subclusters within common Seurat labels (namely B-cells (light/dark green clusters), NK cells (light/dark gold clusters) and erythrocytes (light gray/black clusters)).

To evaluate the biological plausibility of the additional clusters identified by *Hclust-Departure*, we identified differentially transcribed genes using the t-test (cluster size larger or equal to 30) or the Wilcoxon rank-sum test (cluster size less than 30) (Fig. 6). The genes colored in red are statistically significant after FDR adjustment ($p < 0.05$), and have a large mean difference. The genes colored in orange have a significant difference but the mean difference is small. Those colored in black do not differ among clusters. Known cellular identity-specific differentiation markers are annotated by name. Their difference in departure representation is consistent with the existence of two functionally distinct populations as recognized by *Hclust-Departure*.

Fig. 6 panel a depicts two types of DCs corresponding to the coral and blue clusters in Fig. 5. DCs are antigen-presenting cells and are classified into two major subtypes: myeloid DCs (mDC) and plasmacytoid DCs (pDC) [35]. Cluster one downregulates the histocompatibility complex (*HLA*) class II molecules and Cystatin C (*CST3*), *LYZ*, *TMSB4X*; the other does not. Thus, the distribution of biologically defined lineage markers validated this unsupervised clustering result.

Fig. 6 panel b depicts two clusters of B cells (corresponding to the light green and dark green clusters in Fig. 5 panels a and b). B cells are classically known for their ability to produce antibodies, yet they are capable of a variety of functions including antigen presentation, production of several cytokines and the suppression of IL-10 secretion [36]. Comparatively high levels of lineage defining plasma B cell transcripts such as *MZB1* and *FKBP11* and *LTB* (an early B cell differentiating factor) differentiate the two clusters confirming that two clusters, rather than one, was consistent with the known biology.

Fig. 6 panel c focuses on Natural Killer (NK) cells (corresponding to the light and dark gold clusters in Fig. 5 panels a and b). NK cells are one of the major subpopulations of lymphocytes and components of innate immunity. Again key lineage markers were differentially expressed among the two NK cells clusters such as *CD56* and *CD16* [37]. The presence of *ZNF90*, *UBA52* and *FAU* suggests that those cells were in an active transcriptional state. The absence of *TUBB* indicates that the cell was in a state of mature NK cell expression.

Fig. 6 panel d depicts the subdivision of erythroid cells. There are two types of erythroid cells: embryonic and mature. These are traditionally differentiated by the downregulation of several hemoglobin genes including *HBB*, *HBA2* and *HBA1* which are expressed during terminal differentiation [38]. The expression of *YBX1*, a transcriptional factor and *SERBP1*, an anti-apoptotic gene, further support the notion that these cells were in the early stages of erythrocytic development.

In sum, *Hclust-Departure* identifies biologically plausible populations from this complex mixture of cells, establishing equivalent performance to existing scRNA-seq algorithms. It also identifies additional subtypes. Obviously, other algorithms can be tuned to fit previously known subpopulations. However, the choice of correct tuning parameters for those methods is necessarily heuristic, specific to each data set, and not necessarily reproducible or robust. By comparison, *Hclust-Departure* has no tunable parameters, other than the significance level and neither has Sigclust2.

*Hybrid Approach: model departure and Louvain clustering*

A key difference between *Hclust-Departure* and other pipelines is the actual clustering algorithm. We therefore combine the DIPD data representation with the Louvain algorithm as implemented in Seurat.

To validate this combination, we used a different, very complex and very well studied data set with known ground truth. These are the Peripheral Blood Mononuclear Cells (PBMCs) data sets defined by [29]. The Zhengmix8eq data set contains 3,994 cells of eight cell types in equal proportions, some of which are quite distinct and some very similar (Fig. 7 panel a). Unsupervised clustering using Seurat with log-normalized transcription using 15 PCs and resolution parameter 0.8 recapitulate the Fluorescence-Activated Cell Sorting (FACS) labels (Fig. 7 panel b), but miss the distinction between T helper, T regulatory, and T memory cells. *Hclust-Departure* without dimension reduction performs slightly better (Fig. 7 panel c). Table 1 shows the confusion matrix. We also explored the more advanced normalization method SCTransform [18]. This method uses the residuals from Negative Binomial regression with the default parameters maintained for clustering. The results do not differ from the default normalization and are included in Additional file 3. None of the pipelines is completely consistent with the FACS labels in identifying subtypes of T cells. This may be due to the limited accuracy of the algorithms or it may be due to FACS labels not correctly signifying the underlying biological complexity, as T cell differentiation can be very fluid. Finally, DIPD-based data representation combined with Louvain clustering performs better than any of the pure pipelines (Fig. 7 panel d). The hybrid method correctly identifies the T cell subsets and subgroups of monocytes (red cluster). This result suggests that modeling UMI counts by departure from Poissoneity has advantages over other normalization/transformation methods independent of the particular clustering algorithm.

To further define the performance of the hybrid approach, different parameters were explored using either DIPD-based representation ($D$) or log-normalized data as input. These were (a) the number of principal components (15, 20, 25 or 30)

and (b) the resolution parameter in the clustering step (0.6, 0.8, 1.0 and 1.2 for the larger eight cell-type data set Zhengmix8eq; and 0.05, 0.1, 0.2, 0.3, 0.5 and 0.8 for the other two four cell-type data sets Zhengmix4eq and Zhengmix4uneq [29]). These experiments used the full $D$ matrix or the top 2,000 most variable genes. Performance is assessed using the Adjusted Rand Index (ARI) [39] and the purity measure of [40] (Fig. 8). Except for Zhengmix4uneq (Fig. 8, panels b and e), DIPD matrix $D$ as input outperforms Seurat using normalized counts as input; however, there are parameter constellations that lead to dramatic performance degradation independent of the data representation. In sum, DIPD-based data representation $D$ combined with Louvain clustering outperforms other normalization steps for UMI data.

*Further validation of the hybrid approach*

Even though the experiments above point to DIPD-based data representation $D$ and Louvain clustering as the optimal combination, a direct comparison between algorithms that use different data representations and have multiple tunable parameters is difficult using experimental data sets with possibly unknown subpopulations: overfitting cannot be decided on experimental data. An alternate approach is simulation based on theoretical distributions alone. This also is challenging because many aspects of the deep biological variation in scRNA-seq data are unknown and beyond current in silico modeling capabilities. These limitations motivate the use of *crafted experiments*. Here, carefully chosen perturbations are overlaid onto real data. Crafted experiments maintain the complexity of the real data, but control the signal versus noise by considering a range of perturbations from weak to strong. We performed two different types of crafted experiments.

Variation in library size (total UMI counts per cell) is a driver of non-relevant variation in scRNA-seq. To explore this issue we artificially magnified the library size and compared different data representations (Fig. 9 panels a and b). As noted above, many pipelines use multiplication and scaling to adjust for the library size effects. This poses a problem for data containing many zeros. This experiment again used the Zhengmix4eq data. To model library size effects, cells with a large or small library size were perturbed to be even larger or smaller (see Methods for details). We compare data representations from DIPD (yellow), log-normalized counts (blue) and SCTransform (green), all using the Louvain algorithm under the same parameter setting (the number of principal components was set to 15 and the resolution parameter to 0.2). Note that DIPD-based data representation does not implement feature selection, but the other methods select the most variable genes (top 2,000 for log-normalized representation and top 3,000 for SCTransform by default). As before, ARI and purity are used to quantitate performance, and both agree. At $F < 0.5$ (weak signal), all data representations perform similarly. At $F > 0.5$ (stronger signal), performance using log-normalized data declines, whereas SCTransform and model departure remain accurate. These results suggest that log-normalization as the sole pre-processing step is sensitive to library size effects.

Next, we crafted artificial clusters by perturbing some large count genes from the homogeneous luminal epithelial cell line data set (defined in [32]). Artificial clusters were created by adding counts to a sub-matrix of the UMI count data matrix (top 500 genes with the largest total counts across cells and 250 randomly chosen cells (from 541 total)). For each entry of that sub-matrix, random counts from the Poisson distribution with parameter $F \times \tilde{\lambda}_{gc}$ were added to the current UMI count $x_{gc}$, where $\tilde{\lambda}_{gc}$ comes from the two-way approximation (see Methods). Small (or large) values of $F$ indicate weak (or strong) signals. These perturbed cells were regarded as an artificial cluster separated from the remaining cells, where an accurate identification was expected for increasing values of $F$. The random selection was repeated ten times. Again, we used the same parameter settings for all data representations (15 PCs and a Louvain resolution parameter of 0.2).

Fig. 9 panels c and d show the mean ARI and the mean purity with standard deviation. Both measures agree. For $F < 0.5$, none of the data representations distinguish the perturbed cells. For $F > 0.5$, DIPD (orange) identifies more perturbed cells, compared to log-normalization (blue), and SCTransform (green). This may be due to the feature selection step limiting the sensitivity at small perturbations. For log-normalized expression, only 27.2% to 45.6% out of the perturbed 500 genes are in the top 2,000 selected genes. Feature-selection based clustering is not as stable as including all the genes across different randomly perturbed cells, as indicated by the larger standard deviations. The SCTransform (green) performs the worst in this particular experiment. This again seems to be because 36.2% to 45.8% of the perturbed genes are among the 3,000 (default) selected genes for this method. This experiment supports the contention that important, local information may be lost during the feature selection step.

### Discussion

We develop an alternative data representation, DIPD, for scRNA-seq data as well as a clustering algorithm based on this data representation. DIPD is applicable to scRNA-seq data that incorporates experimental UMI correction. With an appropriate number of latent vectors in the GLM-PCA parameter estimation, the IPD statistical framework gives reasonable fits for diverse UMI data sets. Departures from the IPD statistical framework (i.e. DIPD) can be incorporated into existing scRNA-seq analysis pipelines and give improved overall performance independent of the particular clustering algorithm.

Working on the scale of probabilities rather than counts offers numerous advantages. First, due to the characteristics of scRNA-seq data (many zeros and low counts in most matrix entries), working in probability space is a more appropriate way to represent the underlying data structures. The DIPD-based data matrix, provides a useful tool to uncover cell heterogeneity from observed counts into a model departure from the hypothesized Poisson parameter matrix, as input to any subsequent analyses. The large number of zeros in scRNA-seq data, which have been considered in row or column based analyses to be zero-inflation, is more precisely viewed as a large number of very small Poisson probabilities. Similarly, the previously reported over-dispersion is explained by variation in the set of individual Poisson parameters within the framework (Fig. 1).

Implementing Sigclust2 in clustering provides an explicit hypothesis testing for each cluster, which avoids parameter tuning. A direct comparison of different data representations demonstrated that DIPD had an improved performance over conventional log-normalized data (Fig. 7, 8). A hybrid approach combining DIPD with the Louvain clustering algorithm gave the best performance (Fig. 9). Using all the data represented as model departure allowed for the detection of weaker signals compared to feature selection based clustering.

A limitation of this pipeline is computational speed because it uses the full feature set. Computational speed vs. the number of features to be included in the model represents a trade-off of any unsupervised learning approach. It is not specific to this data representation.

At this point, we have only begun to identify biological scenarios that favor this data representation over others. It is necessary to explore additional scenarios where the DIPD and *Hclust-Departure* show differences compared to other approaches. This may identify properties of scRNA-seq data beyond over-dispersion and zero inflation.

The idea of departure based data representation could also be used for other data types based on other distributions, for example, the Assay of Transposase Accessible Chromatin sequencing (ATAC-seq) data based on Binomial distributions.

### Conclusions

Most of the existing scNRA-seq analysis methods suffer from a too crude aggregation at either gene or cell level. We proposed shifting the focus from modeling counts to modeling probabilities and avoided the crude approximations by our IPD statistical framework. We investigated the validity of this model using some carefully designed experiments. As a result, we achieved improved cell clustering performance using a novel data representation based on departures from the estimated Poisson distributions without prior feature selection or manual optimization of hyperparameters. The idea of our DIPD as data representation can also be combined with other clustering methods, such as the Louvain algorithm implemented in Seurat. This novel data representation is useful in better understanding the mechanism of scRNA-seq.

### Methods

#### Data Description

The main performance of the Poisson independent framework for data representation is illustrated using multiple data sets representing different scRNA-seq categories. These are described in the next subsections. They are in increasing order of biological complexity: (i) single cell line data, (ii) three cell line mixture data, (iii) normal human PBMC data, (iv) data from a mouse tissue infected with the human immunodeficiency virus (HIV). The data represented a variety of technical platforms.

*Single clonal cell line data*

To study a scRNA-seq data set which is as homogeneous (and thus Poisson) as possible, single cell line experiments were considered. The first data set is on the experiments of [27]. This data set uses flow-cytometry to place individual cells into wells of a plate. This approach carefully controls the occurrence of doubletons and conversely allowed us to artificially create wells containing doubletons. The experiment is based on two cancer cell lines, which were obtained from human Primary Effusion Lymphoma, called JSC-1 and BCBL-1. These cell lines are clonal and have been in culture for many years. Based on extensive biological characterization each culture is homogeneous, and within a cell line each cell is identical.

The overall experimental design is nested, generating different levels of batch variation. Batch category one represents technical replicates called plates. Cells within a plate are from the same cell line, collected at the same time and hence are homogeneous in that sense. Batch category two represents data of experiment or biological replicates. The full data set contains 10 plates, (1, . . . , 4, 5A, 5B, 6A, 6B, 7, . . . , 10). The data were pre-processed as described in [27]. Specifically, filtering was done such that each cell had greater than 5,000 total UMI counts and greater than 1,500 detected cellular transcripts. Only protein coding transcripts that were detected in more than 0.5% of all cells were retained. The data set used here had a total of 621 cells and 12,689 genes.

This carefully constructed data enabled us to validate the *Poissoneity* under different scenarios, i.e. different degrees of batch variation. The data are summarized in Table 2. For instance, Plates 1 and 2 were from the same cell line but performed on different dates (biological replicates); Plates 3 and 4 also used the same cell line, but were performed on the same date (technical replicates). They were expected to be more similar as technical variation is smaller than biological variation. Data labeled Plate 5A and 5B represent cells where the scRNA-seq libraries from the same cell was sequenced in two independent runs. Thus these were the most similar data sets. The only variation should be due to randomness from the Poisson distribution. Plates 6A and 6B were from an entirely different cell line JSC-1, and were expected to give a radically different expression signature from the BCBL-1 cell line. Plate 8 investigated the impact of doubletons by intentionally putting two cells per well.

*Three cell lines mixture data*

This data set was generated from a mixture of three cell lines by 10X Genomics as in [41] and cleaned by [32]. There are three cell lines in this data set: human dermal fibroblasts-skin, breast cancer luminal epithelial cell line, and breast cancer basal-like epithelial cell line. These were mixed at a ratio of 1:3:6. The cell of origin label for each cell was retained. The data were pre-processed as discussed in [32]. This data set contains 2,609 cells with known labels and 21,247 genes.

*PBMC data*

This scRNA-seq data was generated using 10X Genomics originally from [42]. Cells contained in this data are peripheral blood mononuclear cells (PBMC) from Homo sapiens. The cells were sorted based on cell-surface markers using Fluorescence-Activated Cell Sorting (FACS). Randomly selected cells from this experiment were assembled by [29] as test data sets to measure the clustering performance of different software packages. In particular, three experimental data sets were assembled, each with different mixture characteristics: Zhengmix4eq (4 cell types of equal proportions including 3,994 cells and 15,568 genes) Zhengmix4uneq (4 cell types of unequal proportions as 1:2:4:6, including 6,498 cells and 16,443 genes) and Zhengmix8eq (8 cell types of equal proportions including 3,994 cells and 15,716 genes).

*Multiple cell lineages data*

This data set was based on a study by [28]. This study sampled mouse spleen tissue and obtained scRNA-seq data sets using the 10X Genomics platform. We used one of the mice (Sample A5) which is comprised of 1,476 cells and 12,822 genes. Seurat data cleaning and cell clustering by default parameters were used in the original report and provided computational cell type labels (more details in [28]).

### Existing Methods

We first discuss the GLM-PCA algorithm, which is applied in parameter estimation for our assessment of the IPD framework. Then we give a brief review of the Seurat pipeline, for data pre-processing steps and cell clustering as an example for the state-of-the-art in RNA-seq data analysis.

#### *GLM-PCA algorithm*

GLM-PCA is an algorithm for computing an analog of PCA in the context of generalized linear models (GLM) (see [25] for details). A typical organization for a scRNA-seq data set is a matrix of counts, where columns denote cells (indexed by $c = 1, 2, ..., C$), and rows denote genes (indexed by $g = 1, 2, ..., G$). Let $x_{gc}$ denote one matrix entry, and let $n_c = \sum_g x_{gc}$ denote the total counts for the cell $c$. The GLM-PCA calculation using the Poisson distribution treats the counts as a random variable: $X_{gc} \sim Poisson(\lambda_{gc})$, i.e.

$$P(X_{gc} = x_{gc}) = \frac{e^{-\lambda_{gc}} \lambda_{gc}^{x_{gc}}}{x_{gc}!} \quad (1)$$

A useful model for $\lambda_{gc}$ is

$$\log \lambda_{gc} = \log n_c + \alpha_g + \sum_{l}^{L} \xi_{gl} \rho_{cl}, \quad (2)$$

where $\alpha_g$ is a gene specific parameter, where $\xi_{gl}$ and $\rho_{cl}$ are factor scores and loadings with latent dimension $L$. The scores and loadings have a similar interpretation as in Euclidean PCA, and capture the biological variability after cell and gene specific offsets are removed. The relationships between the Poisson and other count models are considered in [43].

#### *Seurat algorithm*

Seurat (Version 3.1.1, [31]) is an R package developed for scRNA-seq data analysis. It enables users to study the cell-to-cell heterogeneity from transcriptome data. Seurat also integrates diverse types of single cell data sets (see more details in [23, 44, 31]). At each step in the computation pipeline, there are multiple hyperparameters to consider. These provide the users with flexibility, but are selected heuristically. Recommendations for these parameters are arrived at empirically and are varied depending on the input data set. Here we briefly review the standard workflow as described in [28].

**quality control**: Genes with less than three positive counts overall were excluded; cells where the unique gene counts (the number of detected genes) were above 2500

or below 200 were excluded; cells with total mitochondrial gene counts greater than 5% of the overall total were excluded.

**normalization by cell**: The gene expression for each cell ($x_{gc}$) was divided by the cell total counts ($n_c$) and this quotient was multiplied by a scale factor of 10,000 (default).

**transformation**: The natural log transformation was applied.

**feature selection**: The standardized variance (more details in [31]) was calculated for each gene, and the top 2,000 (default) genes with the highest cell-to-cell variation were retained.

**scaling**: The expression of each gene was scaled to have a mean of 0 and variance of 1 across cells. A variation of standard scaling includes regularized negative binomial regression, which is called SCTransform [18].

**linear dimension reduction**: The data was represented by the first 15 principal components obtained by Euclidian PCA.

**clustering**: Cell clustering was done with a graph-based clustering approach using the Louvain algorithm and visualized using t-SNE or UMAP methods.

### Novel Methods

In the following section, we describe the approach for assessment of the validity of the IPD statistical framework. We propose DIPD as a novel data representation, which is a measurement of the relative location of that UMI counts with respect to the independent Poisson distribution at the individual entry level. The cell heterogeneity can be better reflected at the scale of continuous possibilities than in the original scale with excess zeros. Therefore, we further develop a departure-based cell clustering algorithms to identify cell subpopulations.

#### *Independent Poisson statistical framework*

We work with scRNA-seq data with individual matrix entries through an IPD statistical framework, where each matrix entry ($x_{gc}$) is a UMI count indicating expression of gene $g$ for cell $c$. In particular, we model that as a Poisson random variable $X_{gc}$, which is independent over genes and cells. The Poisson probability function is given in equation (1).

In this framework, the maximum likelihood estimate of $\lambda_{gc}$ is the UMI count $x_{gc}$, which is not useful because of the large amount of natural Poisson variation. This motivates combining information and one approach is the GLM-PCA algorithm.

The challenge to measuring the goodness-of-fit is that can not be done using only one data point. We approach this by aggregating matrix entries $x_{gc}$ which have similar Poisson parameters $\lambda_{gc}$, i.e. choosing a reasonable number of entries (in this paper we use 200, which allows assessing the "Poissoneity" without introducing too much variation in the actual underlying parameters) with estimated Poisson parameters closest to some given values, and regard the UMI counts from these 200 entries as independent and identically distributed random samples generated from the Poisson distribution with that parameter. Such nearly homogeneous examples are considered using both Q-Q plots and hypothesis tests. Specifics for measuring "Poissoneity" are described in the next sections.

Note that when using formula (2) to get parameter estimates, the choice of latent dimensions $L$ was important. When $L$ was too small, the model was not flexible enough to appropriately handle biological effects such as cell cycle. So the Poisson distribution did not provide a good fit to the 200 entries. When $L$ was too large, the model was too flexible and was driven by Poisson variation, resulting in overfitting and thus a different poor description of the data. If our underlying IPD framework assumption was correct, there will be a choice of $L$, where we get a good fit of the Poisson distribution. So the existence of such an $L$ was a validation of our underlying IPD framework. We approach this by attempting multiple values of $L$ and assessing if their results were a reasonable fit. This suitable value can be different for different data sets.

#### *Q-Q plot for small discrete counts*

Visualization methods are useful for assessing the "Poissoneity" of scRNA-seq data.

In general, the Q-Q (Quantile to Quantile) plot provides a useful visualization for comparing two distributions. These distributions can be either continuous or discrete, and a common application is to compare a data set represented by its Cumulative Distribution Function (CDF), with a hypothesized probability distribution, also represented by its (theoretical) CDF. The Q-Q plot shows the respective quantiles (the input or argument of the two CDFs) on the vertical and horizontal axes, corresponding to all the probabilities between 0 and 1. The closeness of the graph to the 45° line indicates the closeness of the two probability distributions.

This is illustrated in panel a in Additional file 4 in the case of two very discrete distributions (with very low counts of the type commonly encountered in scRNA-seq data). Using the notation $p_i = P(X = i)$ for the distribution P on the vertical axis, and $q_i = P(X = i)$ for the distribution Q on the horizontal axis. Note that $p_i$ and $q_i$ can either be values from a theoretical distribution such as the Poisson, or can represent empirical probabilities derived from count data as proportions. In this illustration example, define P as $p_0 = 1/3$, $p_1 = 1/2$, $p_2 = 1/6$ and Q as $q_0 = 2/3$ and $q_1 = 1/3$.

Because of the strongly discrete nature of these distributions, the standard Q-Q plot, shown as black dots in panel a in Additional file 4 is quite hard to visually interpret. They do reflect the few integer values taken on by these random variables, but essentially ignore the important probabilities driving the difference between these distributions.

We provided a more informative version of the Q-Q plot by using the idea of *continuity correction*, which provides a useful bridge between continuous and discrete distributions. For example, this idea was the key to the Normal approximation of the Binomial. The main idea was to approximate an integer valued discrete distribution, with a continuous probability distribution, as seen in panel b in Additional file 4. The simple version shown there was a step function, with steps at the half integers, where the height of each rectangle was the corresponding probability. The CDF of a continuity corrected discrete distribution was piecewise linear with knots at the half integers (essentially a linear interpolation), as illustrated in panel c (for the distribution in panel b). The Q-Q plot comparing respective quantiles of the two distributions was shown as the blue curve in panel a. Because both CDFs were piecewise linear, this curve was as well, with knots at the union of the CDF knots.

In the case of checking an empirical CDF against a potential theoretical model CDF, a useful device for understanding the natural variation in a Q-Q plot was the *Q-Q envelope*. This visualization identifies which observed aspects were important and which were artifacts of sampling variation. This idea modeled the hypothesized sampling process by simulating repeated samples of the same size from the candidate theoretical distribution, and overlaying the envelope of resulting CDFs (also using the idea of *continuity correction*). In the case of conventional Q-Q plots (shown as black dots in panel a Additional file 4), this gave a useless visual impression in low count discrete settings. But as seen in Results section, the continuity corrected Q-Q envelopes are very useful.

*Over-dispersion test*

In the case of the Poisson distribution, an insightful test was the dispersion test. An important property of the Poisson distribution was the mean equals the variance. However, many mixtures of Poisson, such as the Negative Binomial, have a variance which was larger than the mean, called *over-dispersion*.

Under the null hypothesis that $H_0 : X \sim Poisson(\lambda)$, we have $E(X) = Var(X) = \lambda$. The over-dispersion alternative is $Var(X) = (1+\alpha)\lambda$, $(\alpha > 0)$. A test statistic was derived (more details in [45]) for measuring this, which is asymptotically normal. This test is conducted using the dispersion test from the R package AER (v1.2-9; [46])

*Zero-inflation test*

A much different departure from the Poisson that can arise in certain applications was *zero-inflation*, where the number of observed zeros was larger than the expected number of zeros. We implemented this test with the R package vcdExtra (v0.7-5; [47]), which was based on a score test proposed by [48] using a test statistic with an asymptotic Chi-square distribution.

*Model departure as data representation*

Again, from our IPD framework, each gene expression measurement for each cell (i.e. each matrix entry) comes from an independent Poisson distribution with parameter $\lambda_{gc}$. A naïve starting point for the application of that framework is viewing cell and gene differences in a purely additive way, i.e. a two-way approximation, expressed as

$$\tilde{\lambda}_{gc} = e^{\mu+\alpha_g+\beta_c}, \tag{3}$$

where $g$ indexes gene and $c$ indexes cell. Of course, there is much richer biological structure beyond this, which we will represent in terms of departures from this approximation of each matrix entry.

*Fitting of a simple two-way approximation* The model (3) is fit to the data using maximum likelihood. In order to make parameter estimation identifiable, restrict that $\sum_g e^{\alpha_g} = G$ and $\sum_c e^{\beta_c} = C$.

There is a closed solution, which is:

$$\begin{aligned}
\hat{\mu} &= \log \frac{\sum_{g,c} x_{gc}}{G \times C} \\
\hat{\alpha_g} &= \log(\frac{\sum_c x_{gc}}{C}) - \hat{\mu} \\
\hat{\beta_c} &= \log(\frac{\sum_g x_{gc}}{G}) - \hat{\mu}
\end{aligned} \tag{4}$$

It's straightforward to prove that the first derivative at parameter estimates defined above are all zero.

We used the above two-way approximation as an initial model, which gave a first order approximation of both library effects and also gene by gene variation. Phenomena, such as cell clustering, were effectively captured by studying the departure from that first order approximation. In other words, features of interest were captured by the difference between the observed UMI counts and the counts expected from the two-way approximation. In particular, the matrix entries that showed significant departure played an important role in cell clustering. The key idea of our departure representation of scRNA-seq data is to replace each count $x_{gc}$ by a number that reflects how well it is explained by the Poisson distribution from the simple two-way approximation. Clustering such numbers is effective at finding structure beyond the two-way fit, such as discriminating cell types. We started by representing departure in terms of where the given count $x_{gc}$ lay in the $Poisson(\tilde{\lambda}_{gc})$ distribution. A naïve approach to this would be to use the UMI count $x_{gc}$ in the CDF of the $Poisson(\tilde{\lambda}_{gc})$ distribution, i.e. $F(x_{gc}; \tilde{\lambda}_{gc}) = P(X \leq x_{gc}|\tilde{\lambda}_{gc})$. While this probability was very effective (i.e. probabilities close to zero or close to one indicate a strong departure) for large values of $\tilde{\lambda}_{gc}$, it was less effective for small values of $\tilde{\lambda}_{gc}$, because the probability had a lower bound of $P(X = 0|\tilde{\lambda}_{gc}) = e^{-\tilde{\lambda}_{gc}} \approx 1$

(as often encountered in scRNA-seq data). This problem was caused by the conventional CDF representation as $P(X \leq x)$. While it was typically not done, CDFs could also be represented as $P(X < x)$, which for our purposes goes too far in the other direction ($P(X = 0|\tilde{\lambda}_{gc}) = e^{-\tilde{\lambda}_{gc}} \approx 0$). Hence, we chose to use the average form of the CDF, i.e.

$$\tilde{F}(x_{gc}; \tilde{\lambda}_{gc}) = \frac{P(X \leq x_{gc}|\tilde{\lambda}_{gc}) + P(X < x_{gc}|\tilde{\lambda}_{gc})}{2}.$$

By doing this, our representation of unexpectedly small UMI counts was nearly 0 and unexpectedly large UMI counts was close to 1.

Another consequence of the generally skewed shape of the Poisson distribution (at least for small values of $\tilde{\lambda}_{gc}$) was that these probabilities tend to be quite asymmetric at the two ends of the distribution. A straightforward device for more balanced treatment of the departures from the Poisson fit was to take the matrix entries to be the logit transform of these CDF based probabilities:

$$D = logit(\tilde{F}(x_{gc}; \tilde{\lambda}_{gc})) = ln(\frac{\tilde{F}(x_{gc}; \tilde{\lambda}_{gc})}{1 - \tilde{F}(x_{gc}; \tilde{\lambda}_{gc})})$$

Since exactly 0 and 1 were not allowed for the logit transformation, set any matrix entries with $\tilde{F}(x_{gc}; \tilde{\lambda}_{gc})$ below $10^{-10}$ as $logit(10^{-10})$, and $\tilde{F}(x_{gc}; \tilde{\lambda}_{gc})$ above $(1 - 10^{-10})$ as $logit(1 - 10^{-10})$.

The logit transformed data takes on very negative (or positive) values if the UMI count is much lower (or higher) than expected from the simple two-way approximation. The collection of cells with such novel data representation can be plugged into a standard clustering algorithm (in this paper we choose hierarchical clustering with Euclidean distance and Ward´s linkage).

*Crafted experiments* For each matrix entry UMI count $x_{gc}$, we calculated the perturbed value by generating a random count from the Poisson distribution with parameter $\left|e^{\hat{\mu}+\hat{\alpha}_g+(1+F)\times\hat{\beta}_c} - \tilde{\lambda}_{gc}\right|$ as $p_{gc}$, where $\hat{\mu}$, $\hat{\alpha}_g$, $\hat{\beta}_c$ and $\tilde{\lambda}_{gc}$ are parameters defined in the two-way approximation and estimated by equation (4). The value for $F$ controls the strength of the library size magnification. Then we perturbed each matrix entry as $(x_{gc} + sign(\hat{\beta}_c) \times p_{gc})_+$, where the subscript of plus denotes the positive part. This magnified the library size effects as the cells with originally positive (or negative) cell effect $\hat{\beta}_c$ become even larger (or smaller).

*Cell clustering algorithm*

The proposed clustering starts with the DIPD-based matrix computed for the complete data set. Hierarchical clustering using Euclidean distance and Ward´s linkage is recommended from a top-down viewpoint. At each step, we re-calculated the two-way approximation again within each subcluster, and the potential for further splitting is calculated using Sigclust2 [30], a method to assess statistical significance at each split based on a Monte Carlo simulation procedure. A non-significant result suggests cells are reasonably homogeneous and may come from the same cell type. In addition, to avoid over splitting, we further require setting a maximum allowable number of splitting steps $J$ (default is 10, which leads to at most $2^{10} = 1024$ total number of clusters) and minimal allowable cluster size $S$ (the number of cells in a cluster allowed for further splitting, default is 10) beforehand. Thus the process was stopped when any of the conditions was satisfied: (1) the split was no longer statistically significant; (2) the maximum allowable number of splitting steps was reached; (3) any current cluster had less than 10 cells. This process was done in a recursive way. Algorithm 1 and Fig. 4 outline the procedure using hierarchical clustering in a recursive way based on departure representation.

We do not need to set the number of clusters beforehand. Thinking of the number of clusters in a multi-scale way as in [32], a coarser scale clustering can be obtained by stopping the clustering process at any stage in between.

**Algorithm 1:** Hierarchical Clustering using DIPD

**Result:** cluster label for every input cell

**Initialize:**

maxSplit $J$ (the maximum allowable number of splitting steps, default 10)

split index $j = 1$

splitResult $R$ ($C \times J$ empty matrix, with cells to cluster as rows, split index as columns)

minSize $S$ (the minimal allowable cluster size, default 10)

complete UMI counts data to cluster $dat_1$

`/* iterate over` $j$ `in a recursive way */`

**Function:**

***hclustDepart(***$dat_j$***,*** $j$***)***

**Input** : UMI counts sub matrix ($dat_j$) with cells in a current cluster; split index $j$

**Output** : splitResult $R$, with $r_{ij}$ denoting the cluster label for the cell $i$ at split step $j$

set $D_j$ to be DIPD-based data matrix calculated from the input UMI count sub matrix $dat_j$

apply hierarchical clustering based on $D_j$ using Euclidean distance and Ward´s linkage use *sigclust2* to find p-value ($p$) for first split

**if** $p > 0.05$ *or* $j > J$ *or number of cells in current cluster* $\leq S$ **then**

output $R$ [all cells, $j$] $= NA$

**else**

split $D_j$ into two clusters ($D_{1j}$, $D_{2j}$) based on hierarchical clustering set $dat_{1j}$ and $dat_{2j}$ to be corresponding UMI counts matrix of two clusters output $R$ [cells in cluster1, $j$] $= 1$; $R$ [cells in cluster2, $j$] $= 2$

***hclustDepart(***$dat_j = dat_{1j}$***,*** $j = j + 1$***)***

***hclustDepart(***$dat_j = dat_{2j}$***,*** $j = j + 1$***)***

**end**

## Declarations

### Ethics approval and consent to participate

Not applicable.

### Consent for publication

Not applicable.

### Availability of data and materials

ScRNA-seq data sets used in this study are all publicly available. The single clonal cell line data is available at `https://bitbucket.org/dittmerlab/scrnaseq_bcbl1/src/master/data/` . The three cell lines mixture data is available at `https://github.com/siyao-liu/MultiK/tree/main/data.` The PBMC data sets can be assessed through the DuoClustering2018 package at `https://bioconductor.org/packages/release/data/experiment/html/DuoClustering2018.html.` The mouse multiple cell lineages data is available at the Gene Expression Omnibus (GSE148796).

### Code availability

R code used to demonstrate the fit of our IPD statistical framework and perform clustering using *Hclust-Departure* is available as an R (CRAN) package and can be accessed from `https://cran.r-project.org/web/packages/scpoisson/index.html.`

### Competing interests

The authors declare that they have no competing interests.

### Funding

This work was supported by PHS grant CA16086, DE018304 to Dirk P. Dittmer. The research of J.S. Marron was partially supported by NSF Grant DMS-2113404. The research of Di Wu was partially supported by University of North Carolina Computational Medicine Program Award 2020.

### Authors' contributions

Conceptualization: YP, JSM, DW, DPD

Software: YP, JTL

Formal analysis: YP, DW, JSM

Investigation: YP, RM, JTL, DPD

Data Curation: YP, JTL

Writing (Original Draft): YP

Writing (Review and Editing): DPD, RM, JTL, DW, JSM

Supervision: JSM, DW, DPD

Funding acquisition: DPD

### Acknowledgements

Not applicable.

**Author details**

[1]Department of Biostatistics, University of North Carolina at Chapel Hill, Chapel Hill, United States. [2]Lineberger Comprehensive Cancer Center, University of North Carolina at Chapel Hill, Chapel Hill, United States. [3]Department of Microbiology and Immunology, University of North Carolina at Chapel Hill, Chapel Hill, United States. [4]Adam School of Dentistry, University of North Carolina at Chapel Hill, Chapel Hill, United States. [5]Department of Statistics and Operations Research, University of North Carolina at Chapel Hill, Chapel Hill, United States.

**Figures**

**Tables**

**Additional Files**

Additional file 1.pdf file

A heatmap view of the data representations of a single cell line data set (Plate 3 [27]). Data representations based on (a) DIPD and (b) Seurat normalized and scaled counts before feature selection. The black colored lines in the sidebars on the right represent the top 2,000 most variable genes kept by the Seurat pipeline. Visually, both data representations demonstrate this data set is homogeneous.

Additional file 2.pdf file

A heatmap view of the data representations of a mixture cell lines data set (three mixture cell lines data [32]). Data representations based on (a) DIPD and (b) Seurat normalized and scaled counts before feature selection. The black colored lines in the sidebars on the right represent the top 2,000 most variable genes kept by the Seurat pipeline. Visually, both data representations effectively demonstrate the differentially expressed genes among the three cell lines. However, highly expressed genes within single cells, as depicted by the bright red spots, may potentially play a role in clustering but many are filtered out by Seurat.

Additional file 3.pdf file

The UMAP plot visualizing the clustering performance in the Zhengmix8eq data set [29] using Seurat SCTransform (15 PCs and resolution parameter 0.8). Each color represents an identified cluster. Similar as the clustering results from Seurat with log-normalized counts, it performs well in identifying the more distinct cell types (NK cells in green, Monocytes in red and B cells in blue), but fails to distinguish T subtypes.

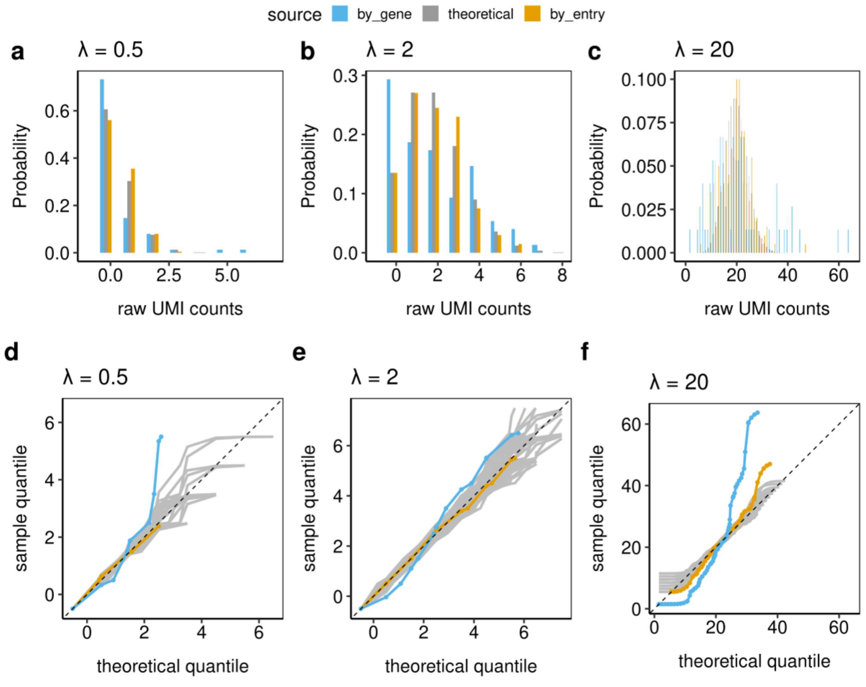

**Figure 1** The distribution histograms (a, b, c) and Q-Q envelope plots (d, e, f) of raw UMI count distributions from 75 biologically clonal cells (Plate 3) as defined in section Methods. The gold bars and lines represent 200 matrix entries with estimated Poisson parameter closest to each $\lambda$; the blue represent the entries from genes whose gene averages are closest to each $\lambda$; and the gray represent the theoretical Poisson distributions. These plots indicate that the IPD statistical framework fits the individual matrix entries well, while working with the gene averages indicates the over-dispersion and zero-inflation may occur.

Additional file 4.pdf file

*Continuity correction* for point mass function (PMF) and Q-Q plot developed for small discrete counts. The black dots in panel a show all the conventional Q-Q points piled up at a few small integers. PMF for distribution P (blue shaded area as *continuous approximation*) is shown in panel b. The CDF for the same distribution P (blue shaded area as *continuous approximation*) is shown in panel c. The blue curve in panel a is the corresponding Q-Q plot comparing two discrete distributions P and Q after *continuity correction* and linear interpolation. It provides a more informative way of comparing distributions with small discrete counts.

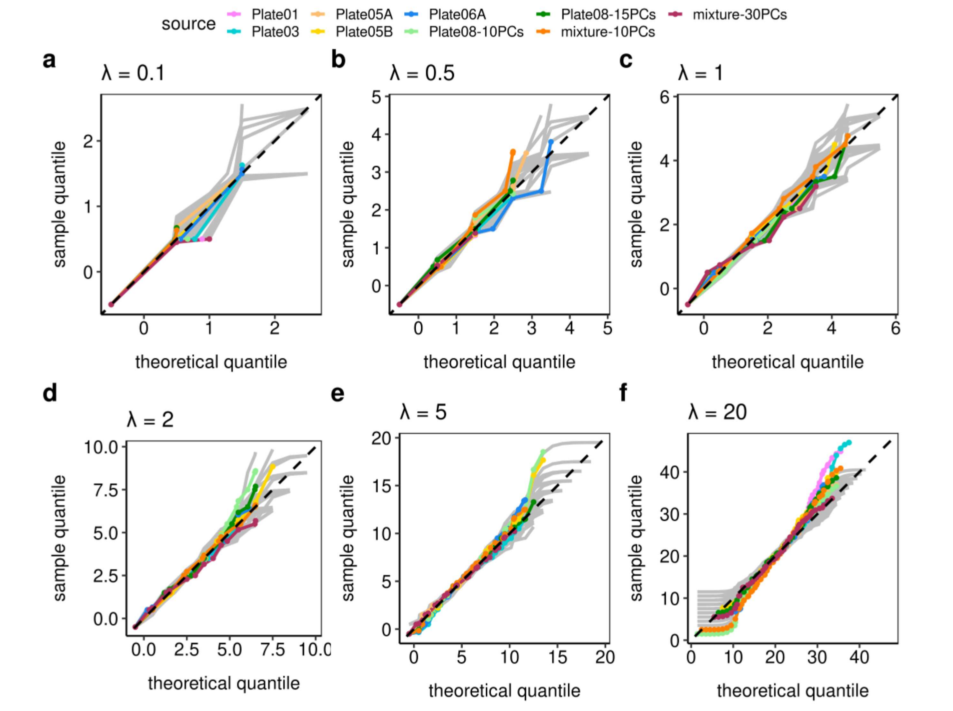

**Figure 2** The Q-Q envelope plots for different Poisson parameters for the different degrees of batch variation. The plots indicate that the IPD statistical framework fits the data well, where most deviations were explained by an inappropriate choice of the number of latent vectors $L$. Plates 1, 3, 5A, 5B are biological replicates of the same clonal cell line. Plate 6A is from a different clonal cell line. Plate 8 has two cells per library. The doublets in Plate 8 required a larger $L = 15$ (dark green) than the default $L = 10$ (light green). The mixture cell lines from Plates 5A and 6A is better modeled by $L = 20$ (red) than $L = 10$ (orange).

**Table 1** Confusion Matrix comparing clustering results with FACS labels

| | Seurat | | | | | | | | |
|---|---|---|---|---|---|---|---|---|---|
| FACS | s0 | s1 | s2 | s3 | s4 | s5 | s6 | s7 | s8 |
| B | 0 | 0 | 0 | 0 | 418 | 0 | 0 | 81 | 0 |
| Monocytes | 1 | 5 | 1 | 547 | 2 | 0 | 0 | 3 | 41 |
| NK | 7 | 6 | 585 | 0 | 1 | 0 | 1 | 0 | 0 |
| T helper | 198 | 180 | 2 | 0 | 0 | 0 | 19 | 0 | 1 |
| T memory | 59 | 394 | 0 | 0 | 0 | 0 | 47 | 0 | 0 |
| Naive Cytotoxic | 26 | 4 | 0 | 0 | 1 | 367 | 0 | 0 | 0 |
| T naive | 472 | 19 | 1 | 0 | 1 | 2 | 3 | 1 | 0 |
| T regulatory | 120 | 230 | 0 | 0 | 0 | 1 | 147 | 0 | 0 |

| | Hclust-Departure | | | | | | | | | | | | | |
|---|---|---|---|---|---|---|---|---|---|---|---|---|---|---|
| FACS | h1 | h2 | h3 | h4 | h5 | h6 | h7 | h8 | h9 | h10 | h11 | h12 | h13 | h14 |
| B | 417 | 34 | 48 | 0 | 0 | 0 | 0 | 0 | 0 | 0 | 0 | 0 | 0 | 0 |
| Monocytes | 0 | 0 | 0 | 0 | 0 | 1 | 0 | 7 | 0 | 0 | 3 | 1 | 558 | 30 |
| NK | 1 | 0 | 0 | 0 | 1 | 0 | 1 | 5 | 0 | 3 | 0 | 589 | 0 | 0 |
| T helper | 0 | 0 | 0 | 214 | 12 | 103 | 16 | 52 | 1 | 0 | 0 | 1 | 0 | 1 |
| T memory | 0 | 0 | 0 | 80 | 11 | 108 | 257 | 28 | 14 | 1 | 0 | 1 | 0 | 0 |
| Naive Cytotoxic | 1 | 0 | 0 | 135 | 240 | 11 | 7 | 4 | 0 | 0 | 0 | 0 | 0 | 0 |
| T regulatory | 0 | 0 | 0 | 164 | 4 | 175 | 17 | 127 | 8 | 0 | 0 | 3 | 0 | 0 |

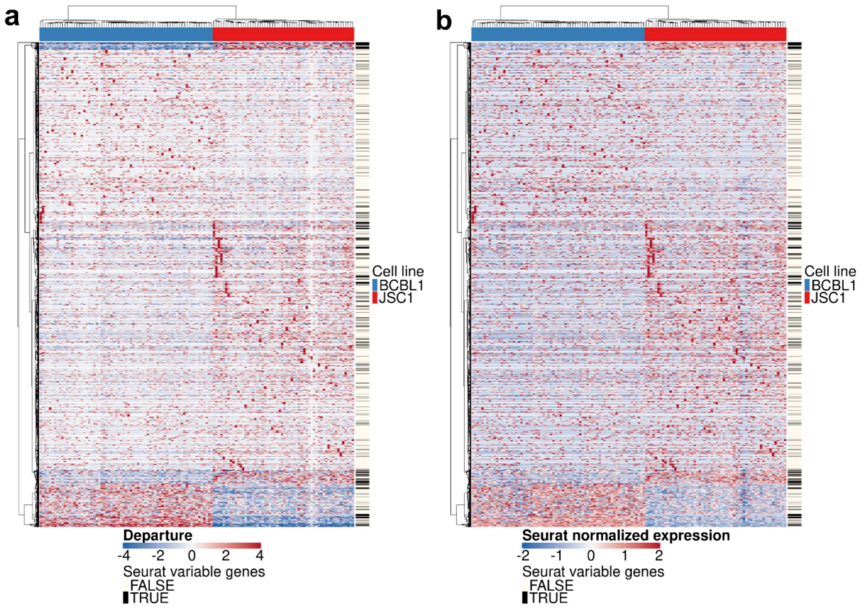

**Figure 3** A heatmap view of the data representations based on (a) DIPD and (b) Seurat normalized and scaled counts before feature selection. The orders of cells and genes for both panels are based on the hierarchical clustering with Euclidean distance and Ward's linkage using model departure. The black colored lines in the sidebars on the right represent the top 2,000 most variable genes kept by the Seurat pipeline. Visually, both data representations effectively demonstrate the differential expressed genes between the two cell lines. However, highly expressed genes within single cells, as depicted by the bright red spots, may potentially play a role in clustering but many are filtered out by Seurat.

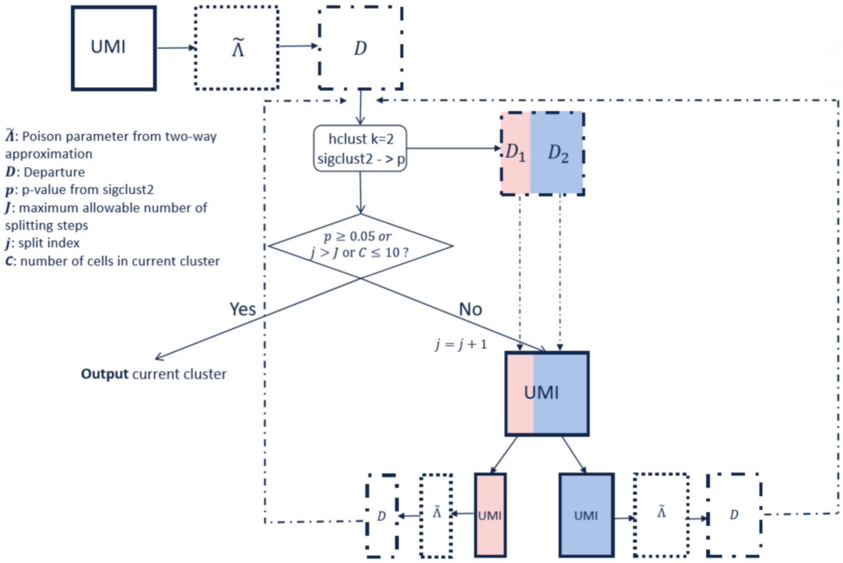

**Figure 4** The *Hclust-Departure* cell clustering workflow. Hierarchical clustering is performed using Euclidean distance and Ward´s linkage in a recursive way.

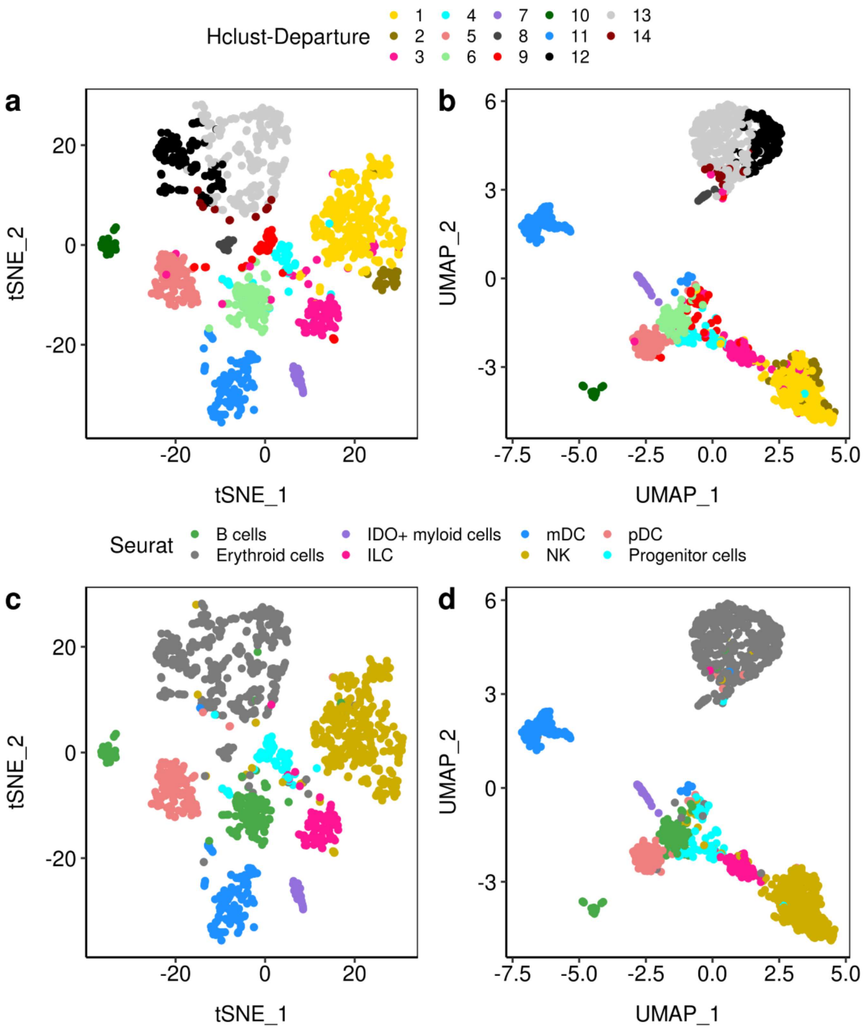

**Figure 5** The t-SNE (panels a, c) and UMAP (panels b, d) visualizations of A5 sample which consists of n=1,476 cells from [28]. The top two panels (panels a, b) were based on *Hclust-Departure* using model departure as data representation. The bottom two panels (panels c, d) were labeled by cell types from the Seurat analysis of [28]. The clusters discovered by *Hclust-Departure* are consistent with those identified by Seurat. Furthermore, *Hclust-Departure* identifies several significant subclusters (namely B-cells, NK cells and erythrocytes).

**Table 2** Summary of plates used

| Plate | Date | Cell Line | Cells Per Well | Cells Per Plate |
|---|---|---|---|---|
| Plate1 | 2018-09-04 | BCBL1 | 1 | 75 |
| Plate2 | 2018-09-11 | BCBL1 | 1 | 80 |
| Plate3 | 2018-09-26 | BCBL1 | 1 | 75 |
| Plate4 | 2018-09-26 | BCBL1 | 1 | 69 |
| Plate5A | 2018-09-26 | BCBL1 | 1 | 71 |
| Plate5B | 2018-09-26 | BCBL1 | 1 | 58 |
| Plate6A | 2018-09-30 | **JSC1** | 1 | 71 |
| Plate6B | 2018-09-30 | **JSC1** | 1 | 59 |
| Plate8 | 2018-09-30 | BCBL1 | **2** | 63 |

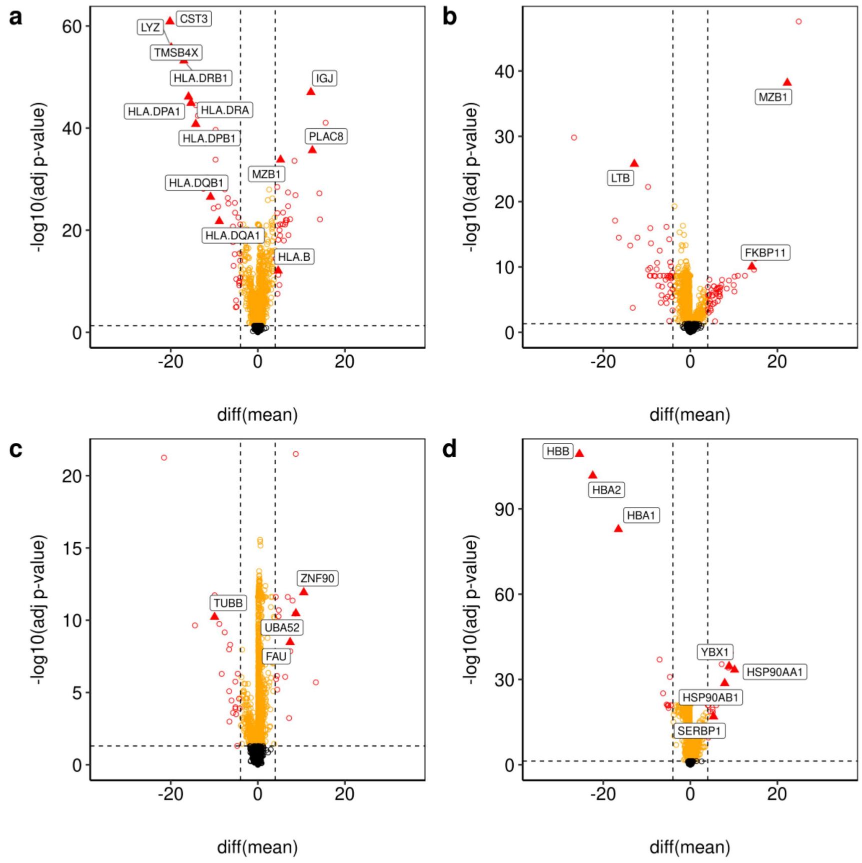

**Figure 6** The volcano plots based on the potential subtypes (depicted in Fig. 5) using differences of mean departure for each gene. Genes are colored as red if the FDR adjusted p-value (vertical axis) is less than 0.05 and the absolute mean departure difference (horizontal axis) is larger than 4 (DE genes); orange if the FDR adjusted p-value is less than 0.05 but the mean departure difference is small; black if the mean departure difference is statistically not significant. Marker genes from DE genes are further triangle annotated and labeled with gene names. (a) Comparing plasmacytoid dendritic cells (pDC) and myeloid dendritic cells (mDC) (coral vs. blue in every panel of Fig. 5); (b) Comparing subclusters within B cells (dark green vs. light green in Fig. 5 panels a and b); (c) Comparing subclusters within NK cells (dark gold vs. light gold in Fig. 5 panels a and b); (d) Comparing subclusters within Erythroid cells (dark gray vs. light gray in Fig. 5 panels a and b)

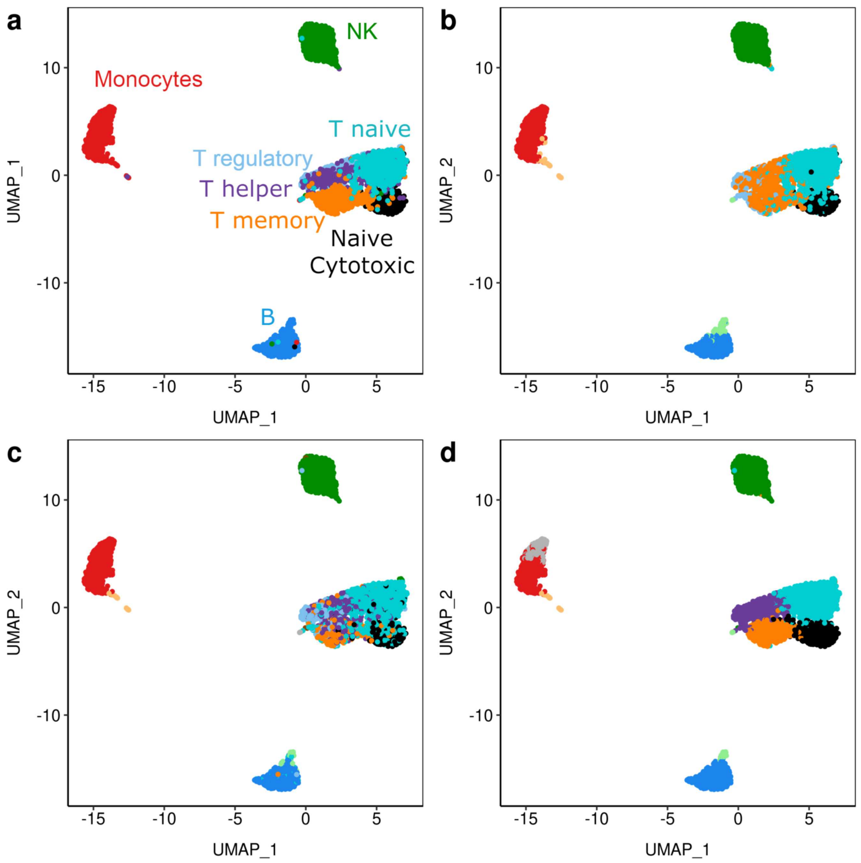

**Figure 7** The UMAP plots comparing clustering performance in the Zhengmix8eq data set [29] using different data representations and clustering methods. Panel a displays the FACS labels we used as a benchmark to measure clustering performance. Both the Seurat pipeline (panel b) and our *Hclust-Departure* pipeline (panel c) correctly identify the distinct cell types but fail to distinguish the subtypes within the T cells. Panel d uses the DIPD-based data matrix as data representation combined with Louvain clustering, which is a more direct comparison with panel b since 15 PCs and a resolution of 0.8 are used in both cases. It improves the original Seurat clustering performance by better distinguishing T memory cells from T helper/regulatory cells.

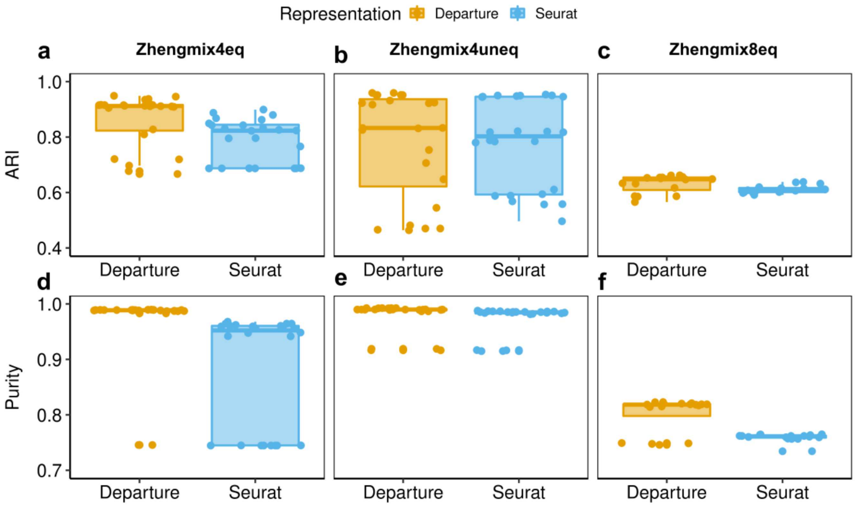

**Figure 8** The boxplots comparing clustering performance using ARI and purity in different data representations. This demonstrates that DIPD-based matrix $D$ as data representation performs better than the Seurat normalized counts in the Zhengmix4eq (four cell types in equal proportions (3,994 cells and 15,568 genes), a and d), Zhengmix4uneq (four cell types of unequal proportions as 1:2:4:6 (6,498 cells and 16,443 genes), b and d), and Zhengmix8eq (eight cell types in equal proportions, c and f) data sets.

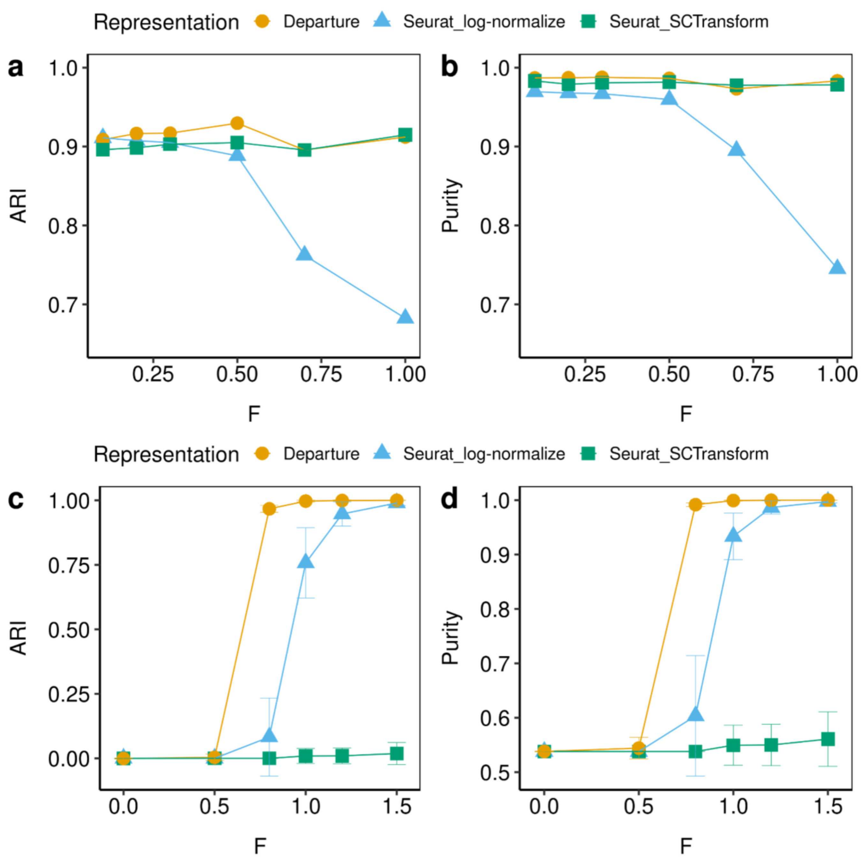

**Figure 9** Comparison of clustering performances using ARI and purity based on different signal strength $F$ (large $F$ means stronger perturbation) in the Zhengmix4eq data set [29] (panels a and b) and luminal epithelial cell line data [32] (panels c and d). Panels a and b magnify the library size effects. The DIPD-based data matrix (orange) as a novel data representation shows a big improvement over the Seurat log-normalized counts (blue) for larger values of $F$, and it performs slightly better than the SCTransform (green). Panels c and d create artificial clusters. The DIPD-based representation (orange) uses information from nearly the full set of genes, and performs the best in identifying artificial clusters for relatively small signals. Both Seurat log-normalized expression (blue) and the SCTransform (green) can lose information during the feature selection step, and result in poor clustering.

## Figures

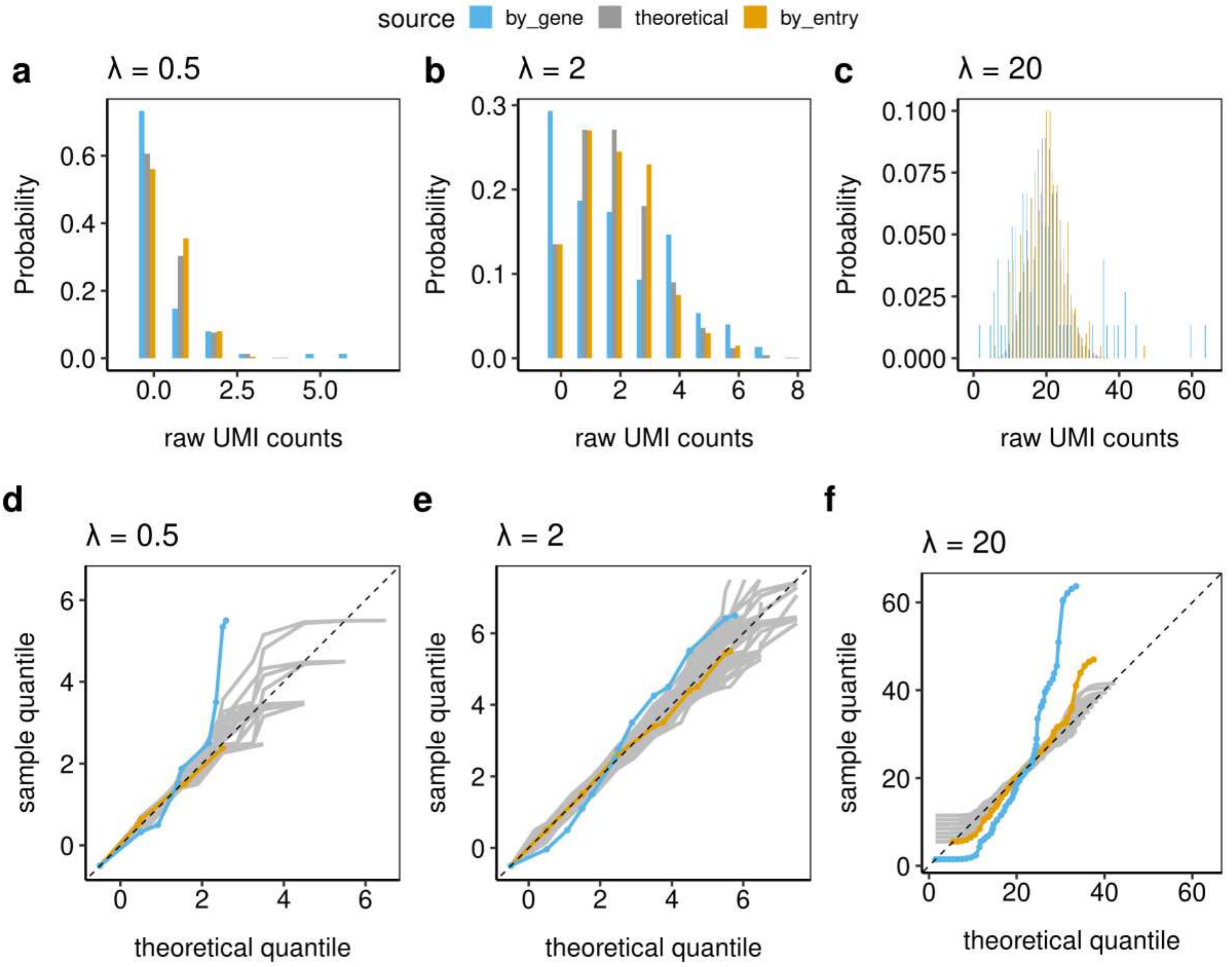

Figure 1

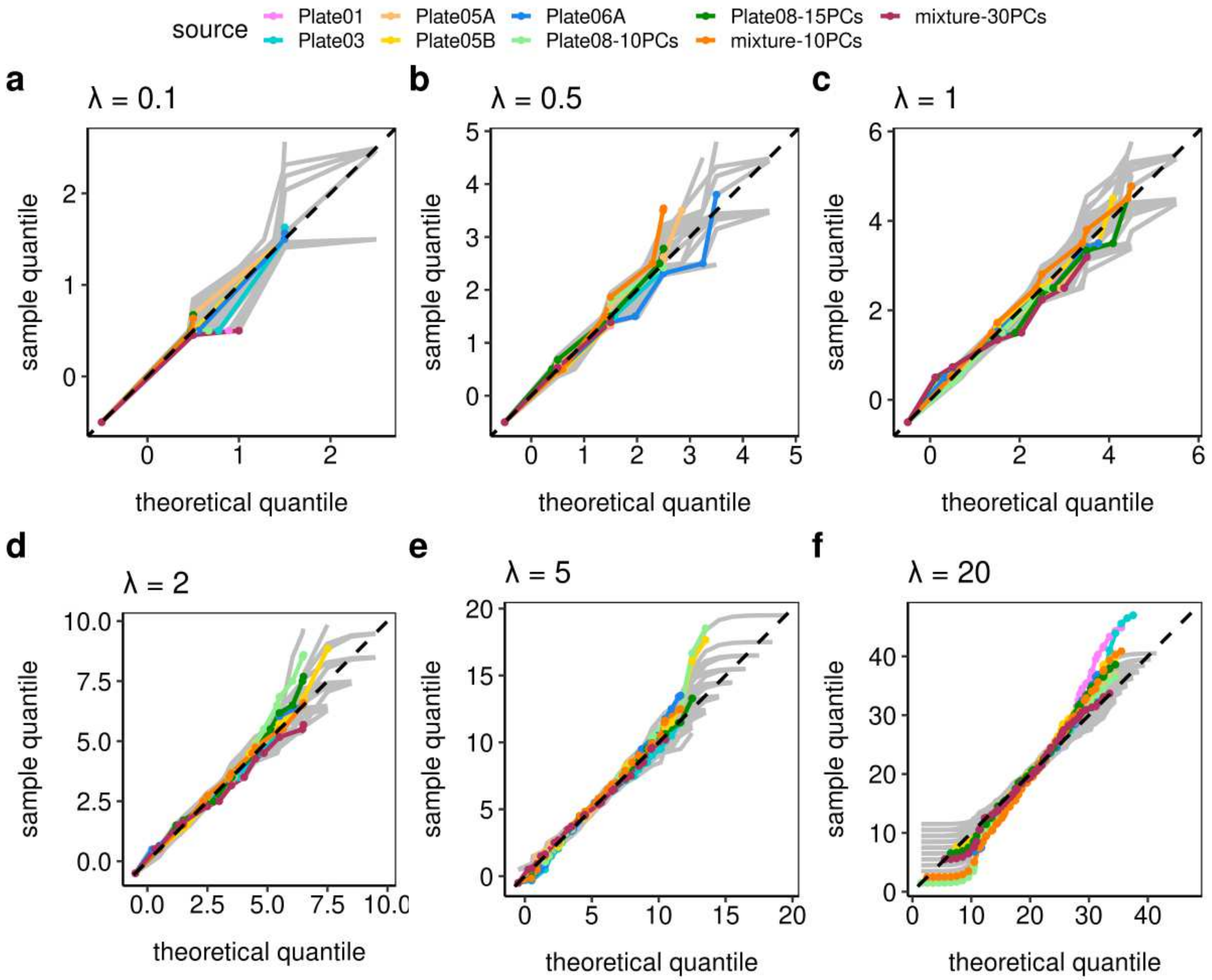

source
Plate01
Plate03
Plate05A
Plate05B
Plate06A
Plate08-10PCs
Plate08-15PCs
mixture-10PCs
mixture-30PCs
a
λ = 0.1
b
λ = 0.5
c
λ = 1
d
λ = 2
e
λ = 5
f
λ = 20
sample quantile
theoretical quantile

Figure 2

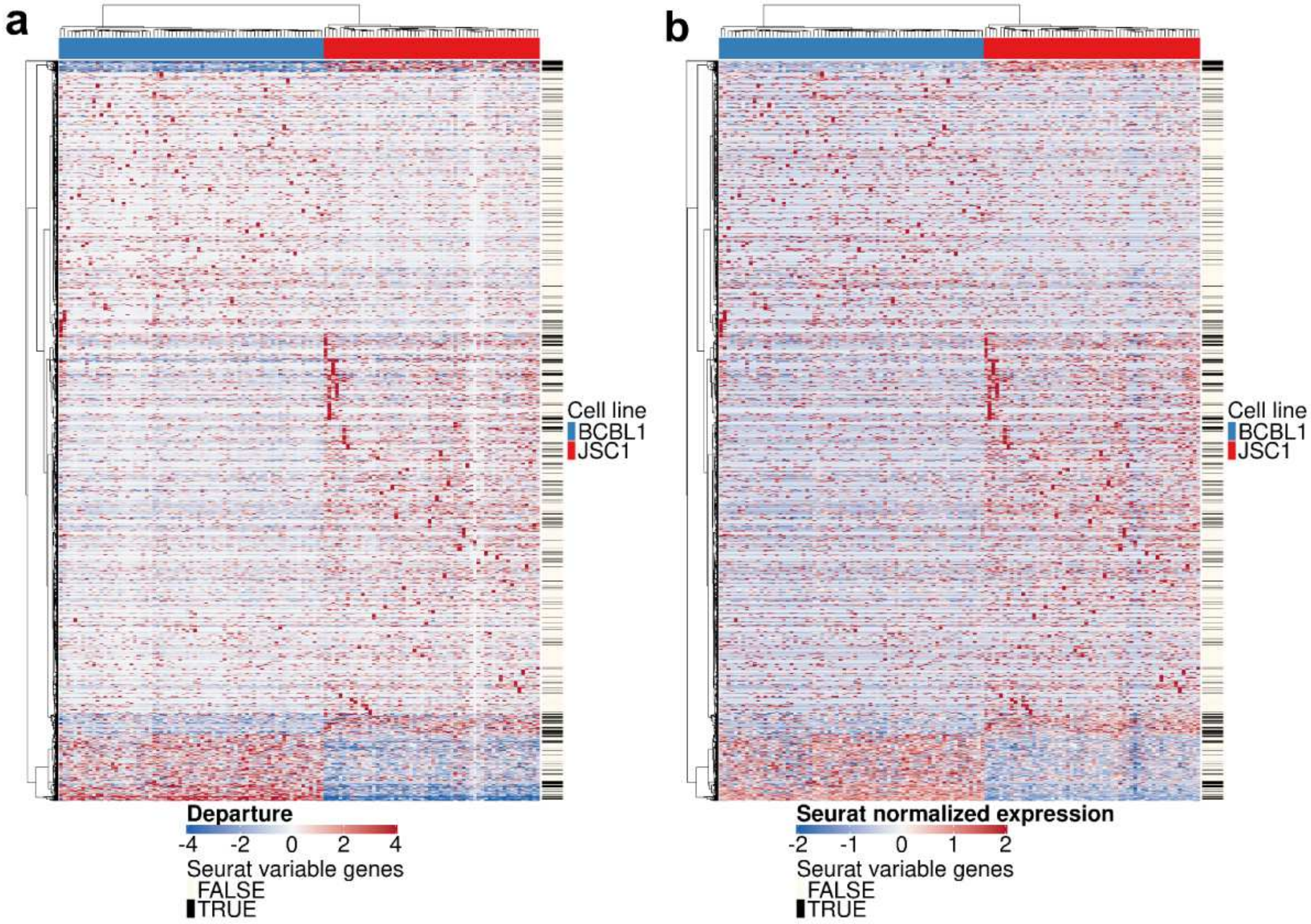

a
b
Cell line
BCBL1
JSC1
Departure
-4
-2
0
2
4
Seurat variable genes
FALSE
TRUE
Cell line
BCBL1
JSC1
Seurat normalized expression
-2
-1
0
1
2
Seurat variable genes
FALSE
TRUE

Figure 3

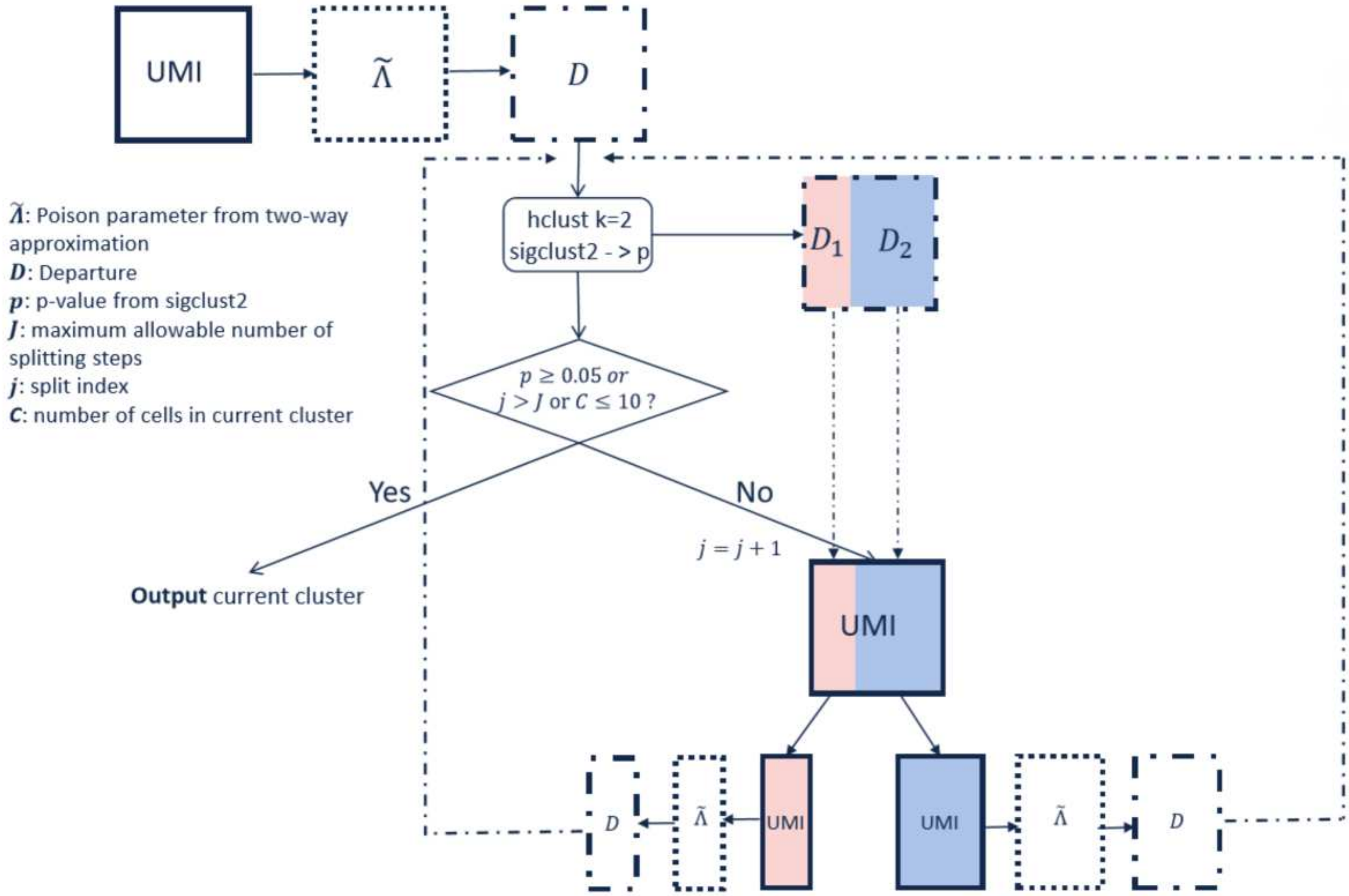

UMI
$\widetilde{\Lambda}$
$D$
$\widetilde{\Lambda}$: Poison parameter from two-way approximation
$D$: Departure
$p$: p-value from sigclust2
$J$: maximum allowable number of splitting steps
$j$: split index
$C$: number of cells in current cluster
hclust k=2
sigclust2 - > p
$D_1$ $D_2$
$p \geq 0.05$ or $j > J$ or $C \leq 10$ ?
Yes
No
$j = j + 1$
**Output** current cluster
UMI
$D$
$\tilde{\Lambda}$
UMI
UMI
$\tilde{\Lambda}$
$D$

Figure 4

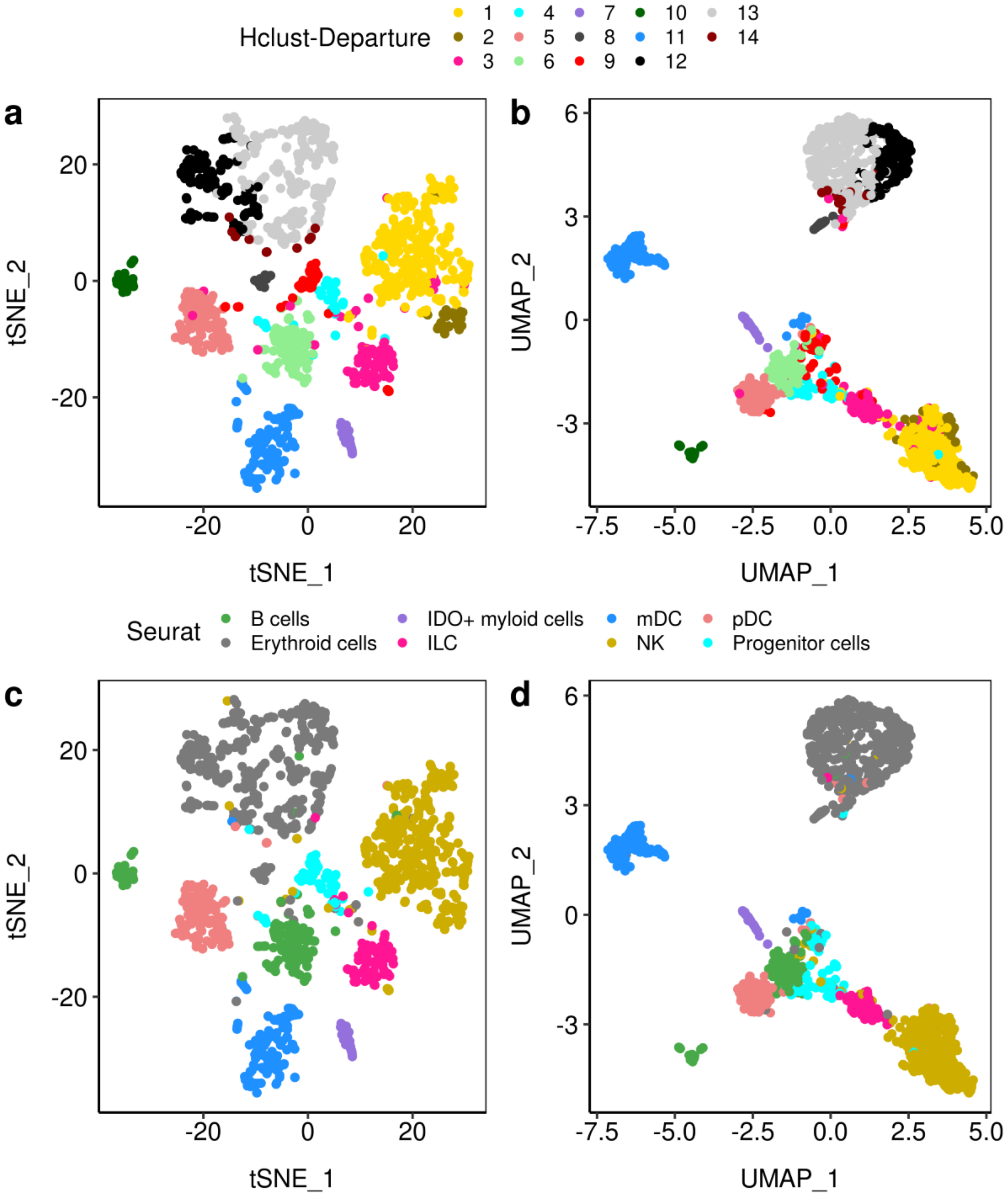

Hclust-Departure
1
2
3
4
5
6
7
8
9
10
11
12
13
14
a
b
tSNE_2
tSNE_1
UMAP_2
UMAP_1
Seurat
B cells
Erythroid cells
IDO+ myloid cells
ILC
mDC
NK
pDC
Progenitor cells
c
d

Figure 5

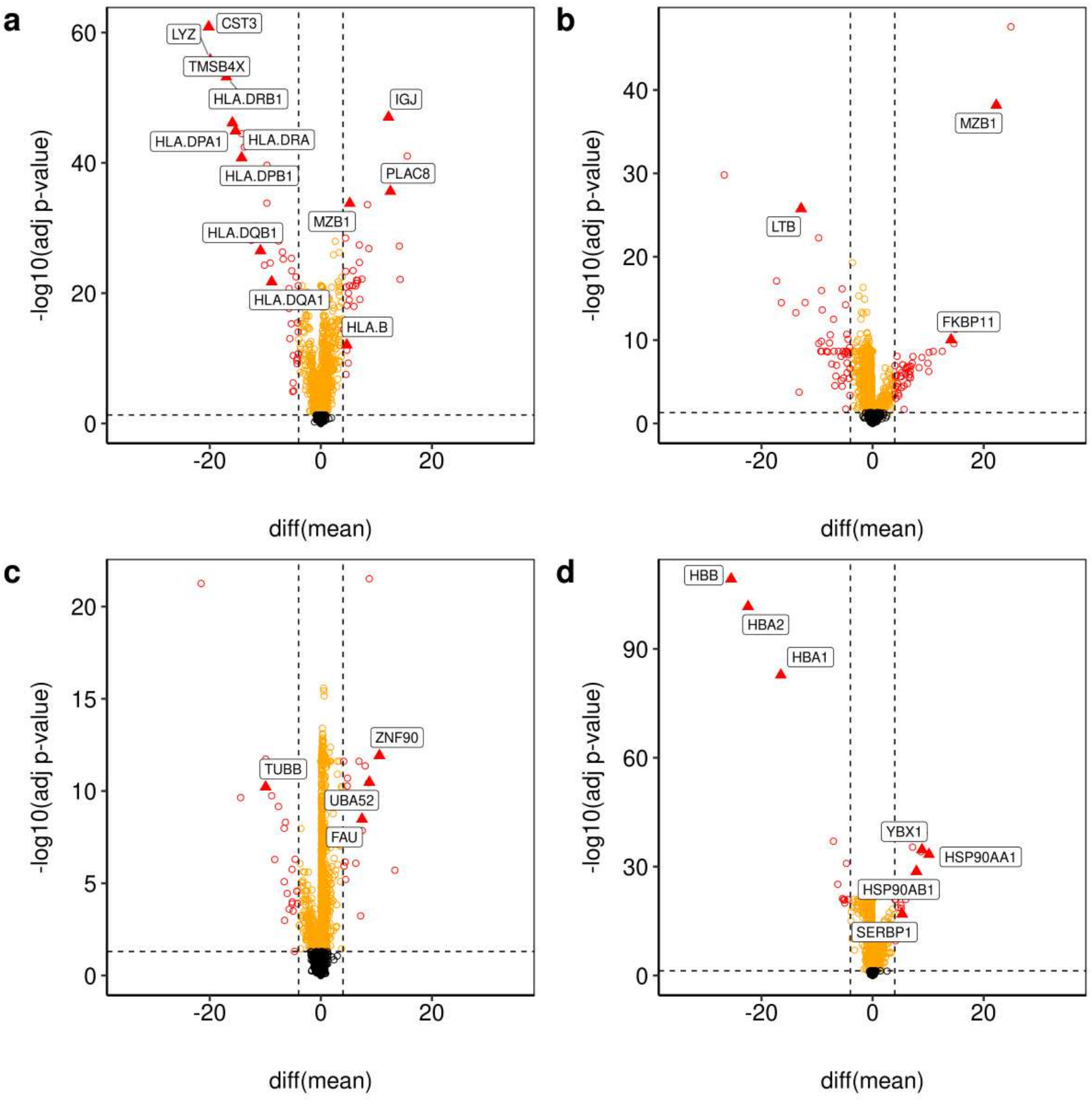

a
CST3
LYZ
TMSB4X
HLA.DRB1
IGJ
HLA.DPA1
HLA.DRA
HLA.DPB1
PLAC8
MZB1
HLA.DQB1
HLA.DQA1
HLA.B
b
MZB1
LTB
FKBP11
c
ZNF90
TUBB
UBA52
FAU
d
HBB
HBA2
HBA1
YBX1
HSP90AA1
HSP90AB1
SERBP1
-log10(adj p-value)
diff(mean)

Figure 6

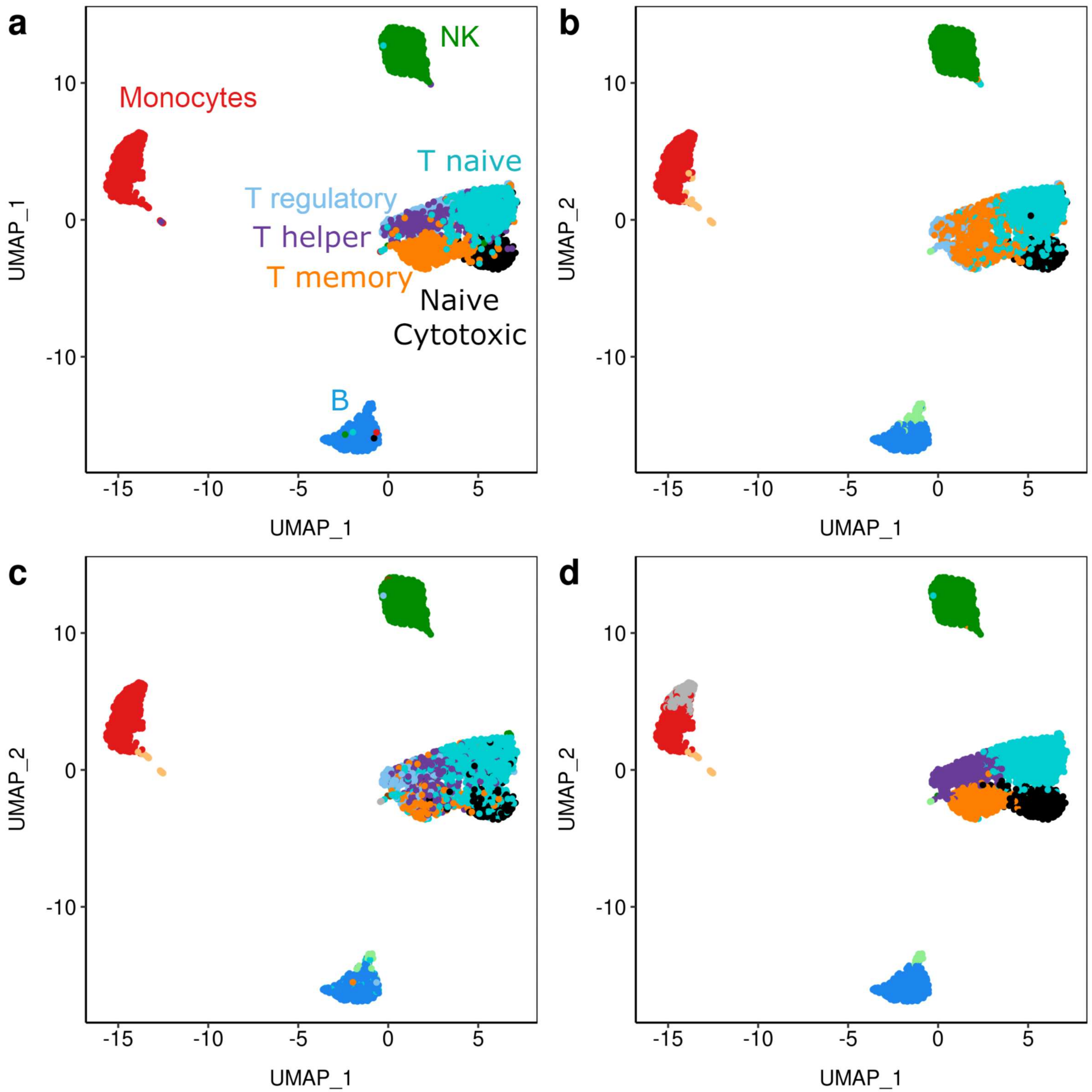

a
NK
Monocytes
T naive
T regulatory
T helper
T memory
Naive
Cytotoxic
B
UMAP_1
UMAP_1
b
UMAP_2
UMAP_1
c
UMAP_2
UMAP_1
d
UMAP_2
UMAP_1

Figure 7

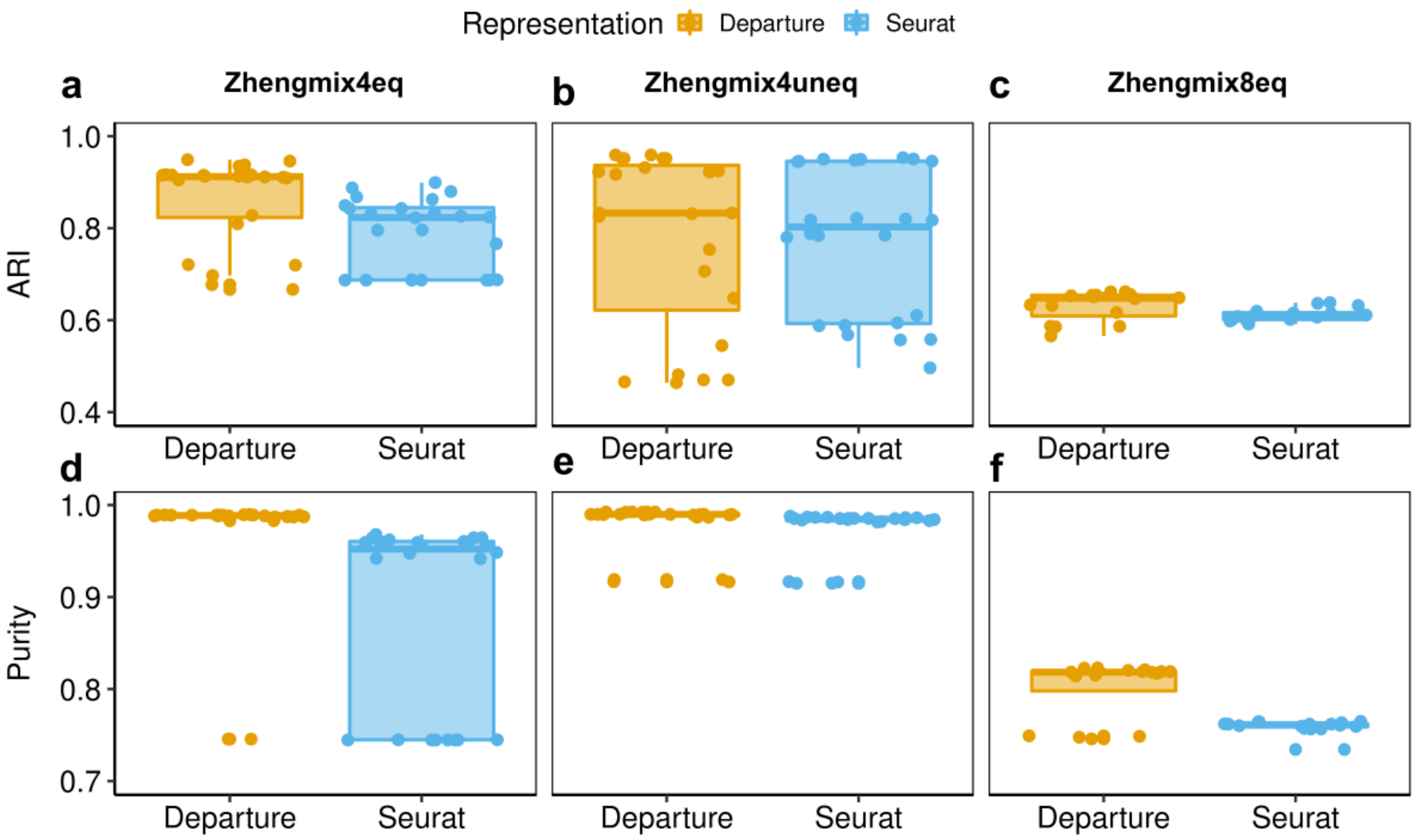

Representation
Departure
Seurat
a
Zhengmix4eq
b
Zhengmix4uneq
c
Zhengmix8eq
ARI
1.0
0.8
0.6
0.4
Departure
Seurat
d
e
f
Purity
1.0
0.9
0.8
0.7

Figure 8

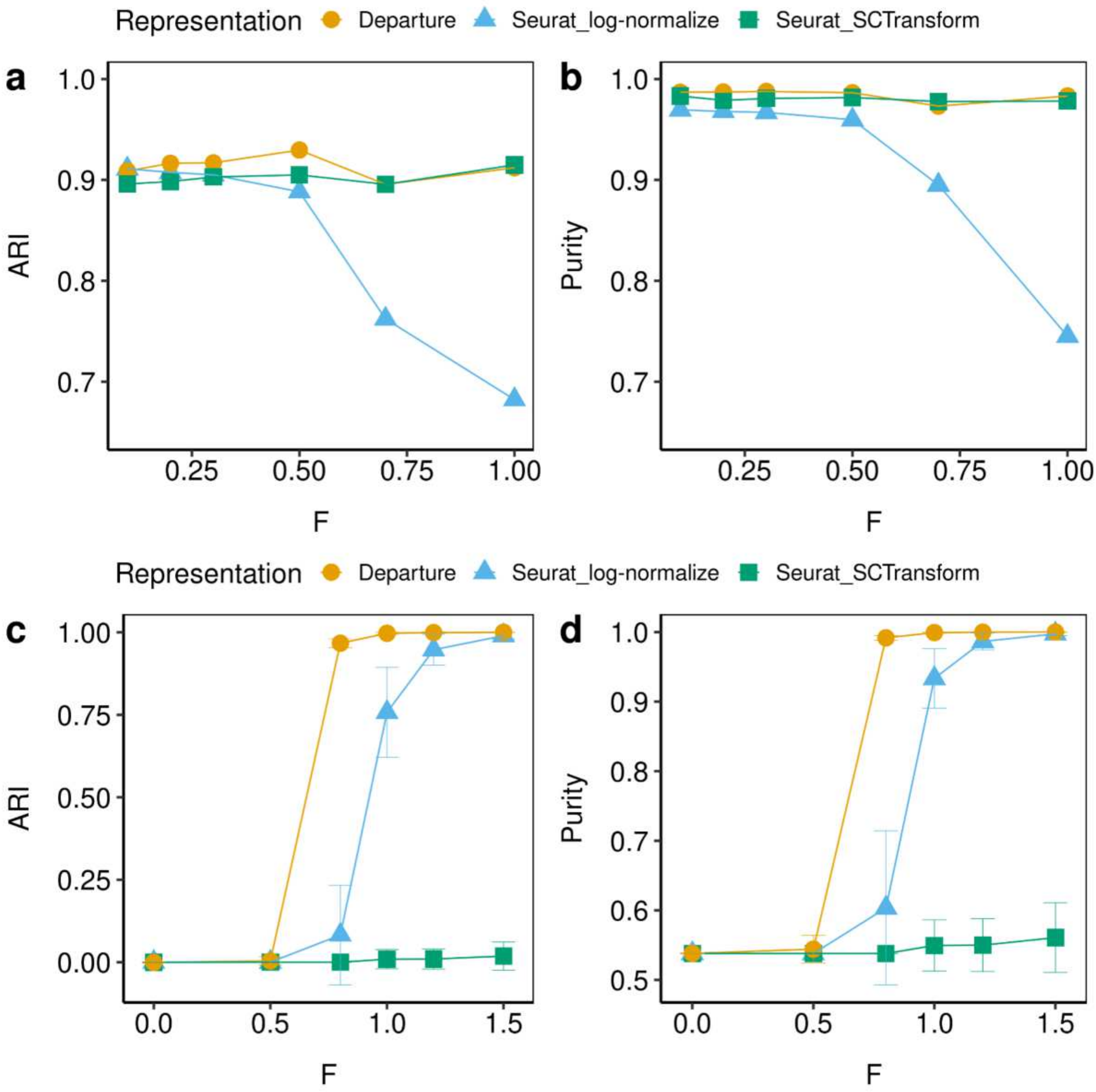

Figure 9

### Supplementary Files

This is a list of supplementary files associated with this preprint. Click to download.

- Table1.docx
- Table2.docx

- Additionalfile1.pdf
- Additionalfile2.pdf
- Additionalfile3.pdf
- Additionalfile4.pdf
